# Structure‐Property‐Application Correlations of Early Transition Metal Chalcogenides: A Dichalcogenide‐Centered Perspective

**DOI:** 10.1002/smll.202508246

**Published:** 2026-02-09

**Authors:** Sachin Jaidka, Aayush Gupta, Daksh Shelly, Yashpreet Kaur, Anushka Garg, Seul‑Yi Lee, Soo‑Jin Park

**Affiliations:** ^1^ Department of Mechanical Engineering College of Engineering Kyung Hee University Yongin Republic of Korea; ^2^ Department of Convergent Biotechnology and Advanced Materials Science Kyung Hee University Yongin Republic of Korea; ^3^ Department of Mechanical Engineering GLA University Mathura Uttar Pradesh India; ^4^ Mechanical Engineering Department Thapar Institute of Engineering and Technology Patiala Punjab India; ^5^ Department of Physics Maharishi Markandeshwar (Deemed to Be University) Mullana Haryana India; ^6^ iCuerious Research Services Mohali Punjab India

**Keywords:** chalcogenides, electrocatalysis, intercalation, optoelectronics, superconductivity

## Abstract

Early transition metal (ETM)‐based chalcogenides constitute a diverse family of layered materials with tunable structural, electronic, and chemical properties. While this materials class includes dichalcogenides, sesquichalcogenides, and polychalcogenides, research efforts and technological applications have been predominantly concentrated on layered transition metal dichalcogenides. This review provides a dichalcogenide‐centered perspective on early transition metal chalcogenides, linking their crystal chemistry and structural polymorphism to functional performance. This review provides a detailed look at various types of ETM‐based chalcogenides, including disulfides, sesquichalcogenides, trichalcogenides, and polychalcogenides, along with their crystal structures and coordination geometries. The review also explains how properties can be modified through doping, intercalation, and strain engineering, and how phase transitions and defects influence their performance. Special attention is given to their use in 2D materials, phase‐change memory devices, and energy‐related applications. By summarizing key experimental findings and structural features, this review offers insight into how ETM‐based chalcogenides can be engineered for better functionality. The combination of their rich chemistry and practical tunability makes them promising materials for next‐generation electronic, catalytic, and energy technologies. Finally, key challenges related to scalability, phase control, interfacial engineering, and environmental impact are critically discussed, and future research is outlined to guide the rational development of next‐generation dichalcogenide‐based technologies.

## Graphical abstract

ETM‐based chalcogenides exhibit rich structural diversity and tunable electronic and catalytic properties. This review critically summarizes structure–property–application relationships in ETM‐based chalcogenides, highlighting recent advances in electronic devices, electrocatalysis, and energy‐related applications, while outlining key challenges and future research directions.

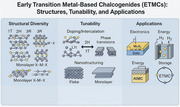

## 1 Introduction

**FIGURE 1:**
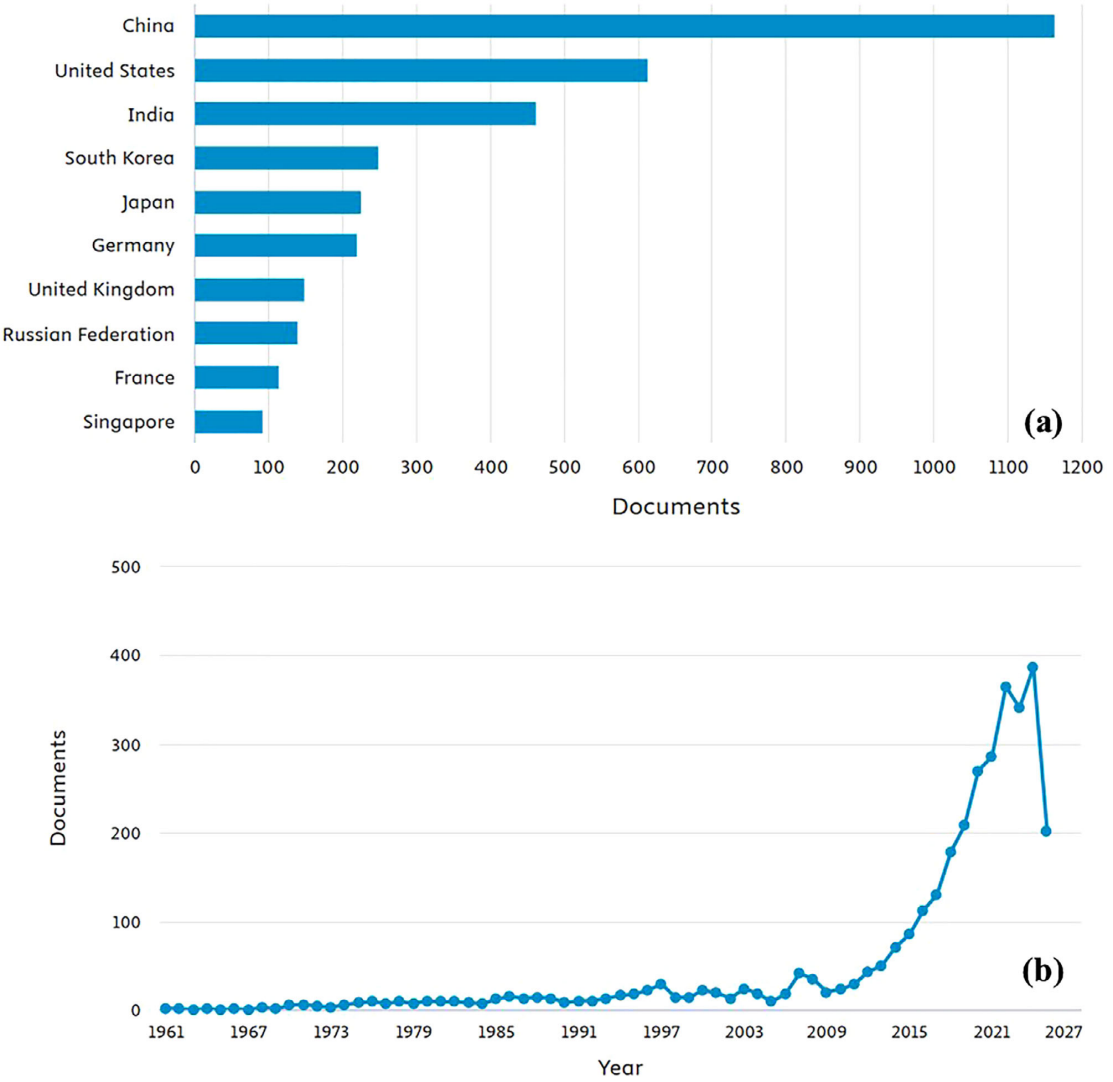
(a) Country wise and (b) year‐wise development of research publications (articles/review papers/conference proceedings/book chapters) across the globe. Data has been collected (as on June 14, 2025) from the SCOPUS database by using ‘transition metal chalcogenides’ as a keyword.

Transition metal chalcogenides (TMCs) are inorganic compounds composed of early transition metals (ETMs) and chalcogen elements. Their general chemical formula is MQ_n_, where M represents a metal from Groups IV‐VII of the periodic table, and Q denotes a chalcogen element such as sulfur (S), selenium (Se), or tellurium (Te). The value of n depends on the metal's oxidation state. These compounds exhibit unique structural and electronic characteristics that make them useful in electronics, catalysis, transistors, photodetectors, DNA sensors, memory devices, and energy storage systems [1–4]. TMCs hold great potential across multiple fields. In energy systems, they are applied in batteries, fuel cells, and other storage devices. In electronics, they are employed in transistors, semiconductors, and integrated circuits. Their catalytic capabilities make them valuable for accelerating industrial and environmental chemical reactions. Furthermore, TMCs are being explored for applications in sensors, superconductors, and biomedical technologies. Their distinct electrical conductivity, thermal stability, and flexibility make them promising candidates for next‐generation materials [5, 6]. Figure 1a illustrates global research activity on “transition metal chalcogenides,” highlighting China as the leading contributor, while Figure 1b shows publication trends from 1961 to the present.

A key research direction involves tailoring the properties of chalcogenides through doping, where selected atoms are inserted into their layered structures. Due to the presence of natural Van der Waals gaps between layers, dopants can be incorporated without significantly disrupting the crystal lattice. By selecting appropriate dopants, one can modify electrical, magnetic, and optical properties, enabling the design of materials for semiconductors, sensors, and batteries. This tunability makes doped chalcogenides a major focus in modern materials research [7]. The foundation for using TMCs in rechargeable batteries was established by Stanley Whittingham, who demonstrated the reversible intercalation of lithium (Li) ions into layered titanium disulfide (TiS_2_). His pioneering work proved that TMCs could act as efficient electrodes, leading to the development of modern lithium‐ion batteries used in devices such as smartphones, laptops, and electric vehicles [8, 9, 10, 11]. ETM chalcogenides are also valuable for studying crystal structure formation and bonding in binary phases. Their diverse atomic arrangements reveal important relationships between structure and properties, helping us understand phase stability and transitions [12].

**FIGURE 2:**
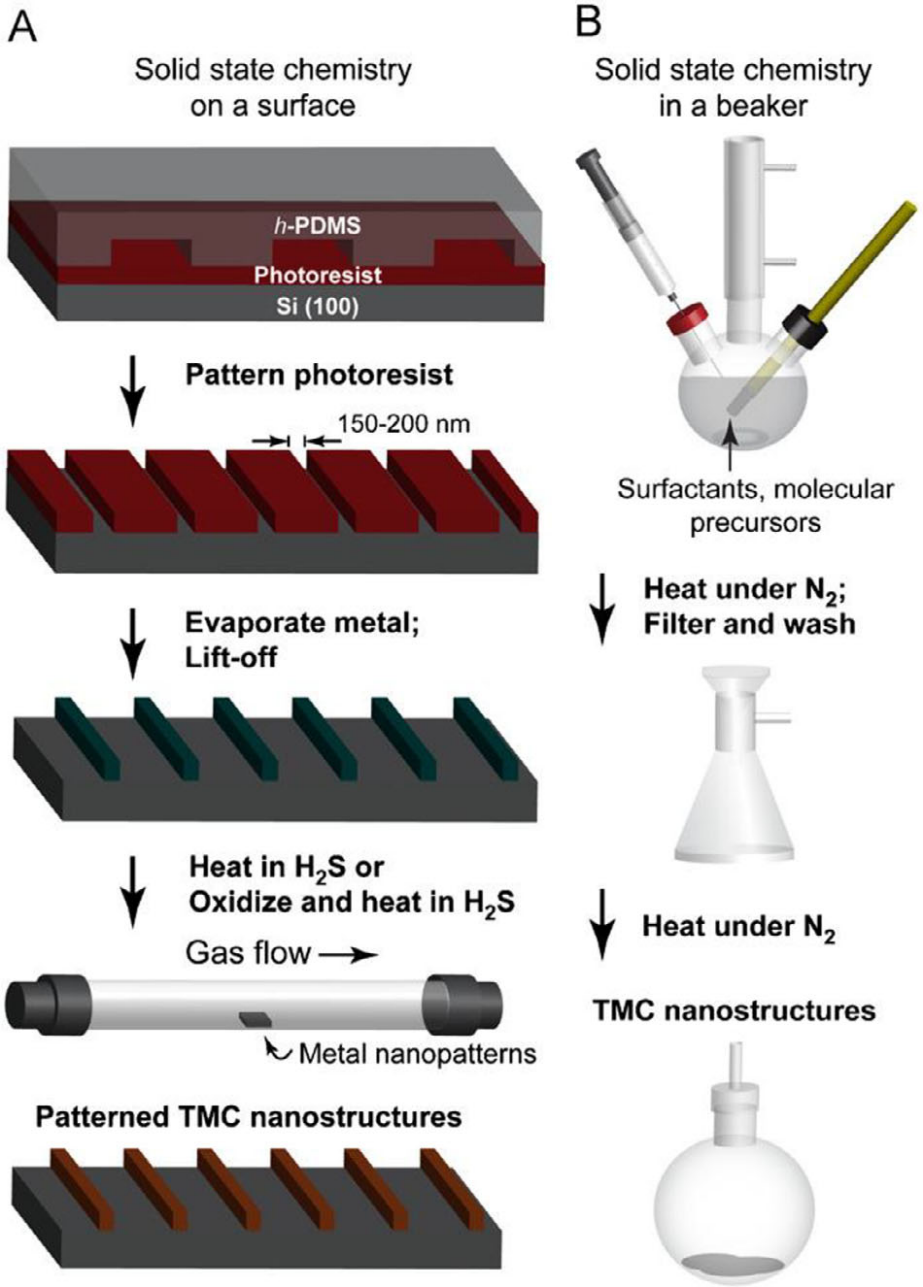
Schematic illustration of (a) chemical nanofabrication method, which begins with photoresist patterning on a silicon substrate, followed by metal evaporation and lift‐off to create nanoscale metal patterns. These patterns are then exposed to a reactive gas environment (H_2_S/oxidation followed by H_2_S treatment) to form patterned TMC nanostructures, and (b) one‐pot fabrication method, where surfactants and molecular precursors are heated under a nitrogen atmosphere. The resulting TMC nanostructures are subsequently filtered, washed, and further heat‐treated under nitrogen to obtain uniform nanoscale products. Reproduced with permission [13]. Copyright 2008, Elsevier.

Figure 2a,b gives the schematics of the chemical nanofabrication and one‐pot fabrication processes for synthesizing nanoscale transition metal chalcogenide materials [13]. It was observed that with chemical nanofabrication, there was an orientational control over nanocrystals within sub‐300 nm patterns of the synthesized MoS_2,_ and free‐standing nanostructures of crystalline NiS_2_ were also formed. Along with this, TaS_2_ nanopatterns were made by the chemical transformation of tantalum oxide templates, as well as crossed line arrays of mixed metal chalcogenide nanostructures were also synthesized successfully. For the one‐pot fabrication method, molecular precursors were used to synthesize 2D NbSe_2_, TaS_2_ and TaSe_2_ nanoplates and 1D NbSe_2_ wires.

**FIGURE 3:**
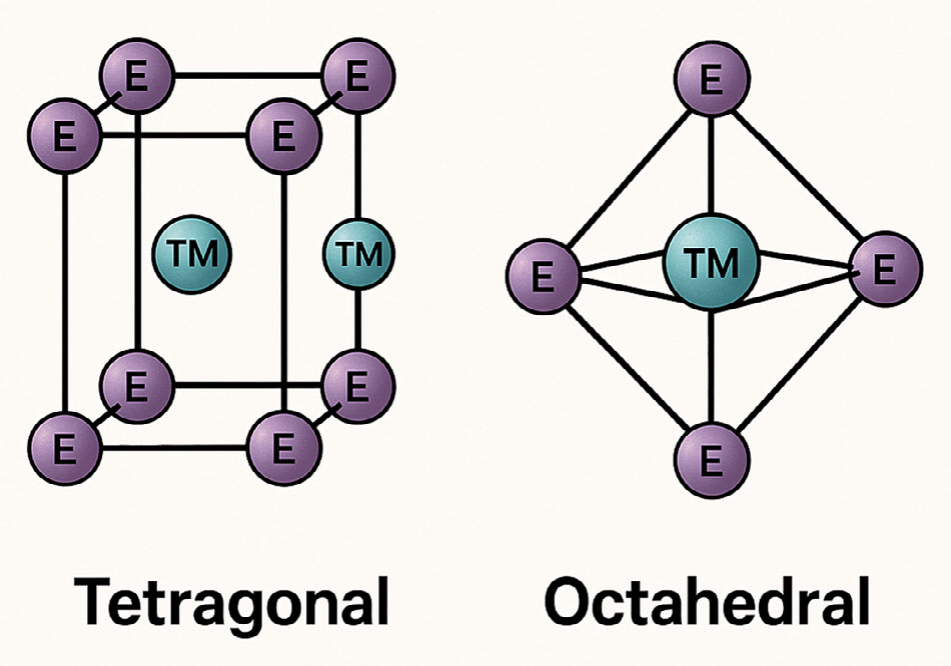
Structural models of early transition metal (ETM) chalcogenides illustrating two common atomic arrangements. In the tetragonal structure (left), each transition metal (TM) atom is coordinated by six chalcogen (E) atoms forming a prism‐like geometry, typically observed in 2H and 3R polytypes. In the octahedral structure (right), the TM atom is surrounded by six chalcogen atoms forming an octahedral geometry, characteristic of 1T phases.

Chalcogenides, typically consisting of one metal atom bonded to two chalcogen atoms, form layered structures with exceptional mechanical and electronic properties. These materials have been synthesized for nearly all transition metals in this group. Among them, molybdenum disulfide (MoS_2_), naturally found as the mineral molybdenite, is well known for its lubricating properties and emerging applications in electronics and catalysis [14]. ETM‐based chalcogenide crystals are composed of vertically stacked 2D layers, approximately 6.5 Å thick [15]. Depending on their chemical composition, these crystals exhibit a range of electrical properties, from semiconducting to superconducting [16]. Single layers can be extracted using micromechanical cleavage, commonly used for graphene production, or through liquid phase exfoliation, a mild solvent‐based technique [17, 18]. The metal atoms in ETM chalcogenides can exhibit octahedral (1T) or trigonal prismatic (2H/3R) coordination, as shown in Figure 3. In the octahedral configuration, each metal atom is surrounded by six chalcogen atoms forming an octahedron, while in the trigonal prismatic structure, the six chalcogen atoms create a prism‐like geometry, influencing the electronic properties [19]. A naturally occurring example is rheniite (ReS_2_), a rare mineral discovered in the Kudryavyi volcano (Kuril Islands, Russia). Containing the valuable element rhenium, it forms under volcanic conditions and is of great interest due to its high melting point and thermal stability [20]. For many years, chalcogenides were considered well‐understood materials. However, the discovery of graphene and its extraordinary properties revived interest in layered materials. It was also realized that chalcogenides shared similar structural features and could exhibit equally fascinating behaviors. This renewed attention led to deeper studies that revealed new electronic and catalytic characteristics, expanding their potential in electronics, energy storage, and catalysis [21].

In recent years, scientists have shown great interest in studying chalcogenides, especially at the nanoscale. The ability to control these properties at such a small scale makes chalcogenides valuable for advanced technologies, including electronics, energy storage, and catalysis. With improvements in synthesis techniques, we can now create high‐quality nanostructured chalcogenides with precise control over their size and shape. This rapid progress in research is opening new possibilities for applications in fields such as quantum computing, flexible electronics, and efficient solar cells [22]. This led to the organization of specialized conferences and the publication of several experimental studies and review articles. These endeavors have unveiled novel, interesting features of chalcogenides and presented original utilizations for these compounds.

## 2 Crystal Structure and Types of ETM‐Based Chalcogenides

The crystal structure of early transition metal (ETM)‐based chalcogenides is primarily determined by the arrangement of metal and chalcogen atoms, leading to various structural types. These materials commonly adopt layered structures, where transition metal atoms are sandwiched between chalcogen layers, forming either trigonal prismatic or octahedral coordination. Depending on the stacking sequence, they exhibit different polytypes, such as 1T (octahedral coordination), 2H (trigonal prismatic coordination with hexagonal symmetry), and 3R (trigonal prismatic coordination with rhombohedral symmetry). ETM‐based chalcogenides include materials like titanium disulfide (TiS_2_), zirconium diselenide (ZrSe_2_), and hafnium telluride (HfTe_2_), which are known for their unique electrical, optical, and catalytic properties [23, 24].

### 2.1 ETM Chalcogenides

Layered chalcogenide materials made from early transition metals have a unique and interesting crystal structure. These materials are built from repeating layers, and each layer consists of three atomic sheets stacked together in the order of chalcogen‐metal‐chalcogen (X‐M‐X). In this formula, “X” stands for a chalcogen atom like sulfur (S), selenium (Se), or tellurium (Te), while “M” stands for a transition metal such as titanium (Ti), molybdenum (Mo), or tungsten (W) [25, 26]. These layers are not connected by strong chemical bonds but are held together by weak van der Waals forces. This means that although the bonds within each layer are strong and stable, the layers can easily slide over one another or be separated with little force. The combination of strong in‐plane bonding and weak interlayer coupling gives ETM chalcogenides a rare balance of mechanical stability and structural tunability, making them ideal for applications in flexible electronics, photocatalysis, and nanoelectromechanical systems. Within the X‐M‐X layers, the metal atoms (M) can be arranged in different geometries depending on the material. The two common arrangements are trigonal prismatic coordination and octahedral coordination. In trigonal prismatic coordination, each metal atom is surrounded by six chalcogen atoms placed in a triangular prism shape. This structure is found in materials like molybdenum disulfide (MoS_2_) and tungsten disulfide (WS_2_), which are widely studied for their semiconducting properties. In octahedral coordination, the metal atom is also surrounded by six chalcogen atoms, but they are arranged in an octahedral shape. These structure‐property relationships offer a powerful means of engineering electronic behavior simply by tuning atomic coordination. This coordination is observed in materials like titanium disulfide (TiS_2_), vanadium disulfide (VS_2_), etc., which often exhibit metallic behavior. It has been observed that the electronic configuration, such as d^0^, d^1^, d^2^, or d^3^, of the metal cation strongly influences phase stability and electronic characteristics. For example, niobium disulfide (NbS_2_), with a d^1^ configuration, can exhibit different structural and electronic behaviors compared to zirconium disulfide (ZrS_2_), which has a d° configuration [27, 28, 29, 30].

Another important feature of these structures is their ability to allow atoms, ions, or even small organic molecules to insert themselves between the layers (a process known as intercalation) [31]. It can occur without destroying the basic structure of the material, which means the X‐M‐X layers remain intact, and only the van der Waals gaps are used for insertion. This structural flexibility makes these materials useful for various chemical and electrochemical applications. For example, TiS_2_ can be intercalated with lithium ions (Li^+^), which is a key process in lithium‐ion batteries. Similarly, organic molecules like pyridine can be inserted into MoS_2_, creating new hybrid materials with tailored properties for sensing or catalysis. Layered ETM chalcogenides are widely recognized as excellent materials for creating nanosheets. Among them, molybdenum disulfide (MoS_2_) is one of the most studied examples. These materials can be broken down into ultrathin layers or sheets using methods like ultrasonic dispersion, exfoliation, and chemical vapor deposition (CVD). These techniques help separate the individual layers without damaging the structure, making them ideal for use in nanotechnology and electronics. ETM‐based chalcogenides such as MoS_2_, tungsten disulfide (WS_2_), niobium disulfide (NbS_2_), and tungsten diselenide (WSe_2_) all share a similar structure [27, 28]. In these compounds, a single layer of positively charged metal atoms (called cations) like Mo, W, or Nb is sandwiched between two layers of negatively charged chalcogen atoms (called anions) like sulfur (S) or selenium (Se). This forms what is often described as a “sandwich” or “trilayer” structure. The metal atoms are arranged in a hexagonal pattern, and the chalcogen atoms sit above and below, creating a stable and symmetric layered arrangement [29, 30]. The capacity to produce high‐quality monolayers with controllable thickness has propelled ETM chalcogenides to the forefront of 2D materials research. One of the most interesting features of these materials is the space between the layers, called the interlamellar space, and this can be used to modify or enhance the properties of these materials by inserting atoms or molecules into this space.

**FIGURE 4:**
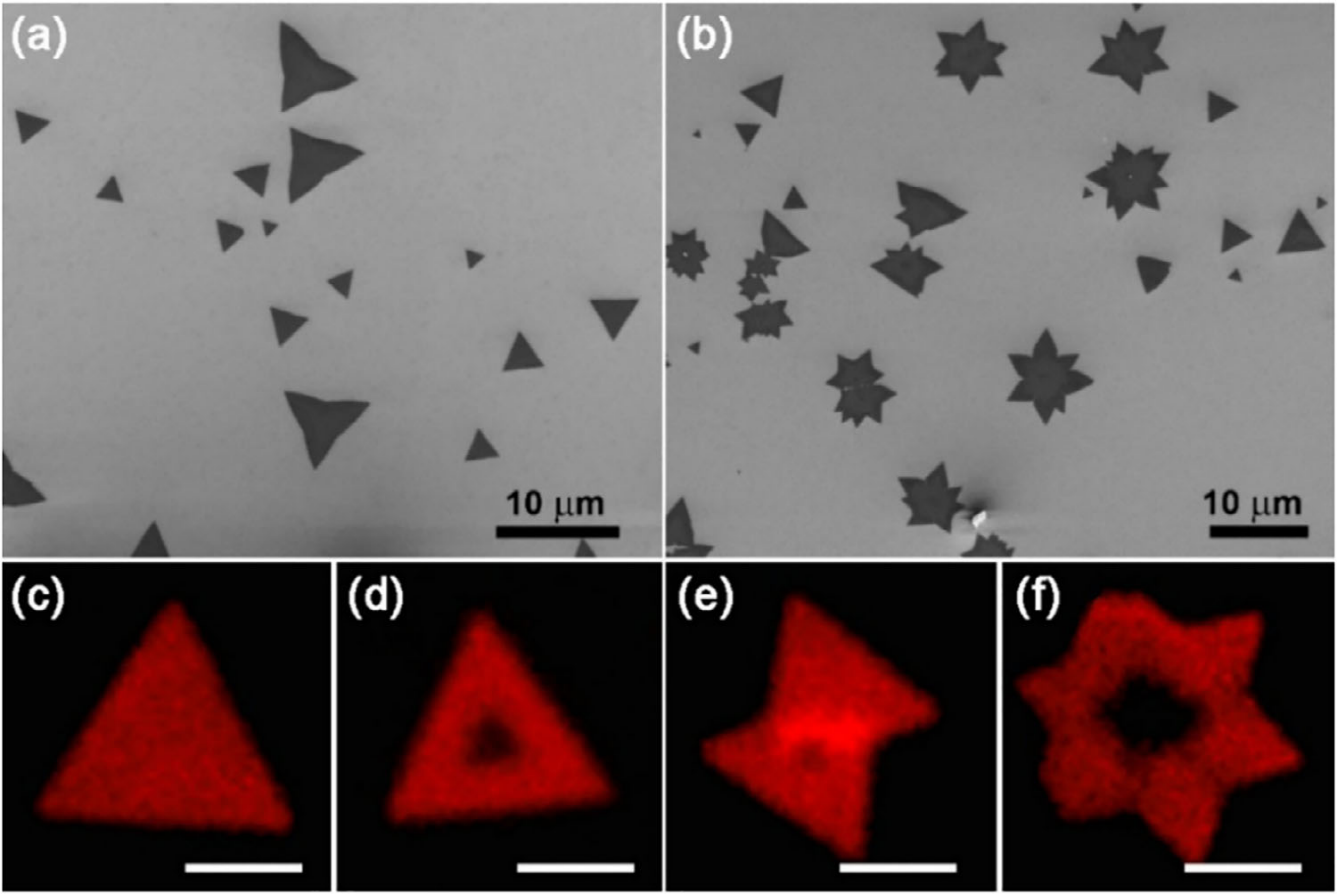
ETM‐based chalcogenide monolayer islands with various morphologies: (a, b) scanning electron microscopy (SEM) images of MoS_2_ monolayer islands synthesized by heating MoS_2_ powders in the presence of S vapor at atmospheric pressure at 700°C, and (c–f) fluorescence images of MoS_2_ monolayers with different morphologies. Reproduced with permission [32]. Copyright 2014, AIP.

Similarly, doping by adding small amounts of other elements can also change their electrical, optical, or catalytic behavior, such as intercalating lithium ions into MoS_2_ can improve its performance as a battery material [33]. These changes can make the material more conductive, more chemically reactive, or even give it magnetic properties, depending on what is added. This flexibility makes ETM‐based chalcogenides very attractive for a wide range of advanced applications. Apart from 2D sheets, several other morphologies of MoS_2_ have also been reported, which were prepared by CVD technique. Perylene‐3,4,9,10‐tetracarboxylic acid tetra potassium salt (PTAS) is an effective seed material for growing MoS_2_ and WS_2_ monolayers [34]. Depending on the growth kinetics and the chalcogen content during CVD, it was possible to develop triangular islands, continuous films, butterfly‐like monolayers, David stars, and other polygonal monolayers, as shown in Figure 4. It is important to note that the number of layers significantly affects the fluorescence intensity. For instance, the indirect bandgap of few‐layered MoS_2_ (e.g., the nucleation center of the triangle shown in Figure 4d) will quench the fluorescence. However, the fluorescence signal can be enhanced near the tilt boundary (Figure 4e) and reduced near the mirror boundary (Figure 4f) [32].

**FIGURE 5:**
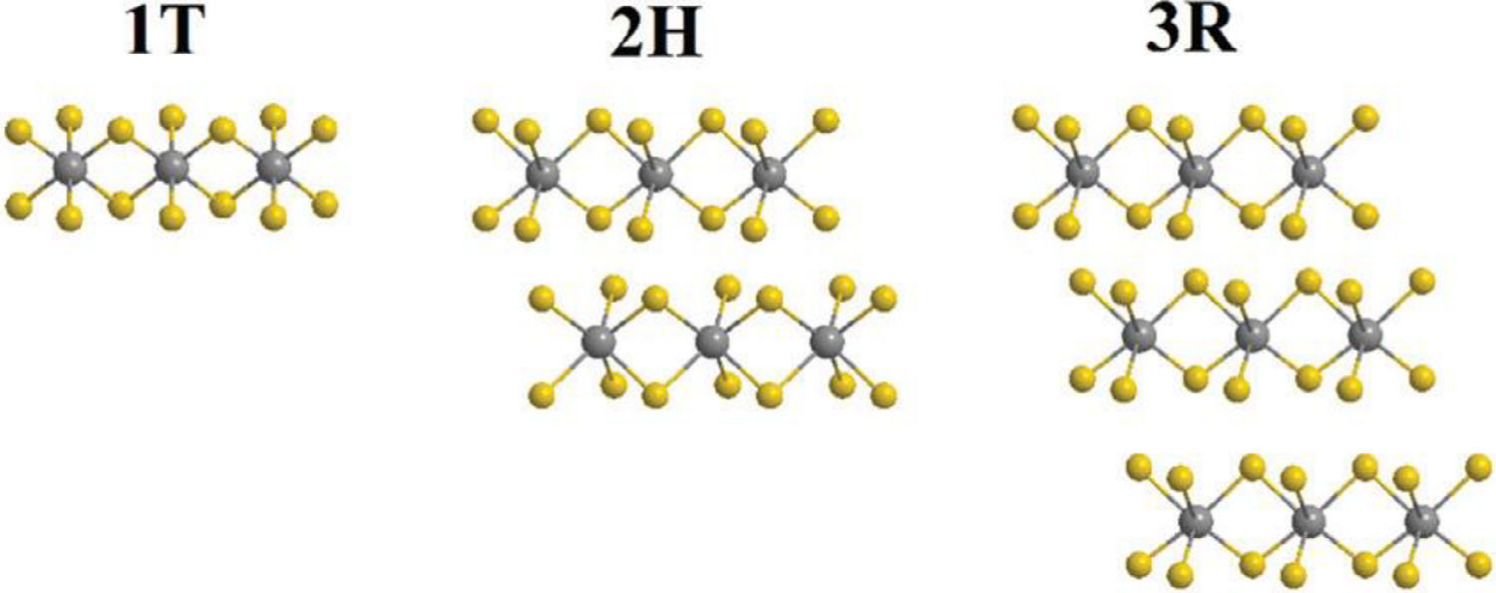
ETM‐based chalcogenides can stack in different ways, creating common structures called 1T, 2H, and 3R (grey spheres‐transition metal atoms & yellow spheres‐chalcogen atoms). Reproduced with permission [19]. Copyright 2021, RSC.

Most ETM‐based chalcogenides have layered crystal structures where metal atoms are positioned between layers of chalcogen atoms. Depending on how these layers stack together, different polytypes arise. The most common polytypes include 1T, 2H, and 3R structures, as shown in Figure 5. The 1T crystal structure is a specific arrangement found in some transition metal chalcogenides, where the metal atoms are surrounded by chalcogen atoms in a particular geometric pattern. In this structure, each metal atom is positioned at the center of an octahedral coordination, meaning it is bonded to six chalcogen atoms that form an octahedron around it. This octahedral shape ensures a stable and symmetrical arrangement of atoms, influencing the material's electronic properties. The layers of atoms in the 1T structure stack in a tetragonal symmetry, which means they follow a repeating square‐like pattern when viewed in 3D space. This unique stacking arrangement allows for strong interactions between layers, impacting how electrons move through the material. One of the most significant characteristics of the 1T structure is its metallic or semi‐metallic behavior. The way the atoms are arranged allows electrons to move easily, giving the material good electrical conductivity. Because of this, materials with a 1T structure are particularly useful in applications where electrical conductivity is required, such as in electronic devices and catalytic reactions.

A well‐known example of a material with a 1T structure is titanium disulfide (TiS_2_). TiS_2_ is widely studied for its potential use in batteries, especially lithium‐ion batteries, due to its ability to store and transfer electrical charge efficiently. The combination of high conductivity and stable structural properties makes the 1T structure an essential component in advanced materials research. In contrast, the 2H structure is one of the most common crystal structures found in ETM‐based chalcogenides. In this structure, each metal atom is surrounded by six chalcogen atoms, forming a trigonal prismatic coordination. This means that the metal atom sits inside a prism‐like arrangement of chalcogen atoms, where the three atoms on the top layer align directly with those on the bottom layer, creating a stable and well‐ordered structure. The layers of these prisms stack together in a hexagonal symmetry, meaning that when viewed from above, the atomic arrangement appears in a repeating hexagonal pattern. One of the key characteristics of the 2H structure is its semiconducting behavior. This means that materials with this structure can conduct electricity under certain conditions, making them highly useful for electronic applications. The semiconducting nature arises from the specific way the atoms are arranged, which affects how electrons move through the material. Because of this, 2H‐structured materials are widely used in devices that rely on controlling electrical signals, such as transistors, solar cells, and light‐emitting diodes.

**FIGURE 6:**
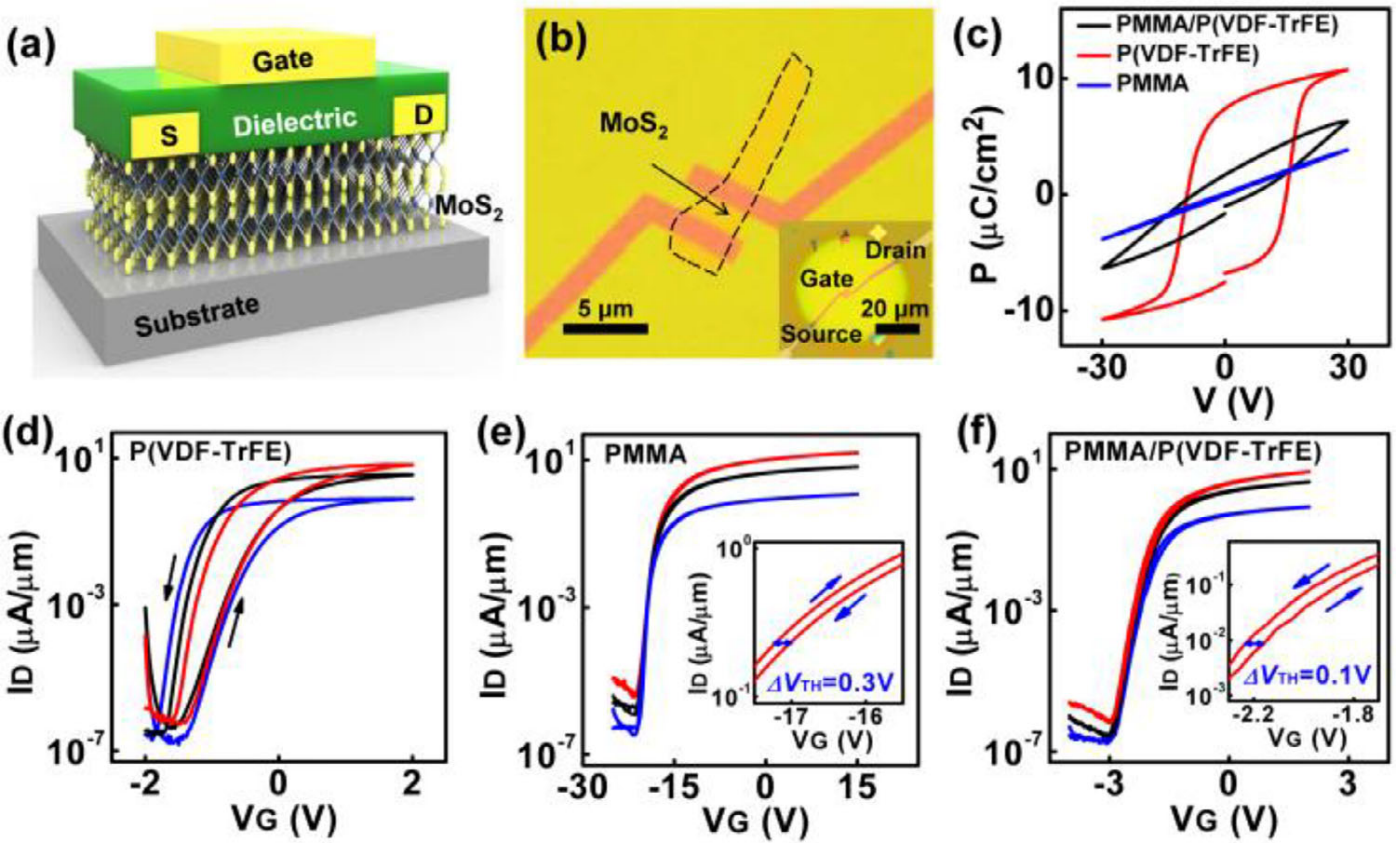
(a) Schematic of the MoS_2_ transistors with P(VDF‐TrFE), PMMA, and PMMA/P(VDF‐TrFE), (b) optical micrograph of the MoS_2_ transistor, (c) PV loops of the dielectrics, and (d)‐(f) typical transfer curves of the MoS_2_ transistors with different gate dielectrics. The red, black, and blue lines correspond to V_D_ = 1 V, 0.5 V, and 0.1 V, respectively. The insets are the zoomed‐in curves of the transfer characteristic with V_D_ = 1 V. Reproduced with permission [35]. Copyright 2020, IEEE.

**FIGURE 7:**
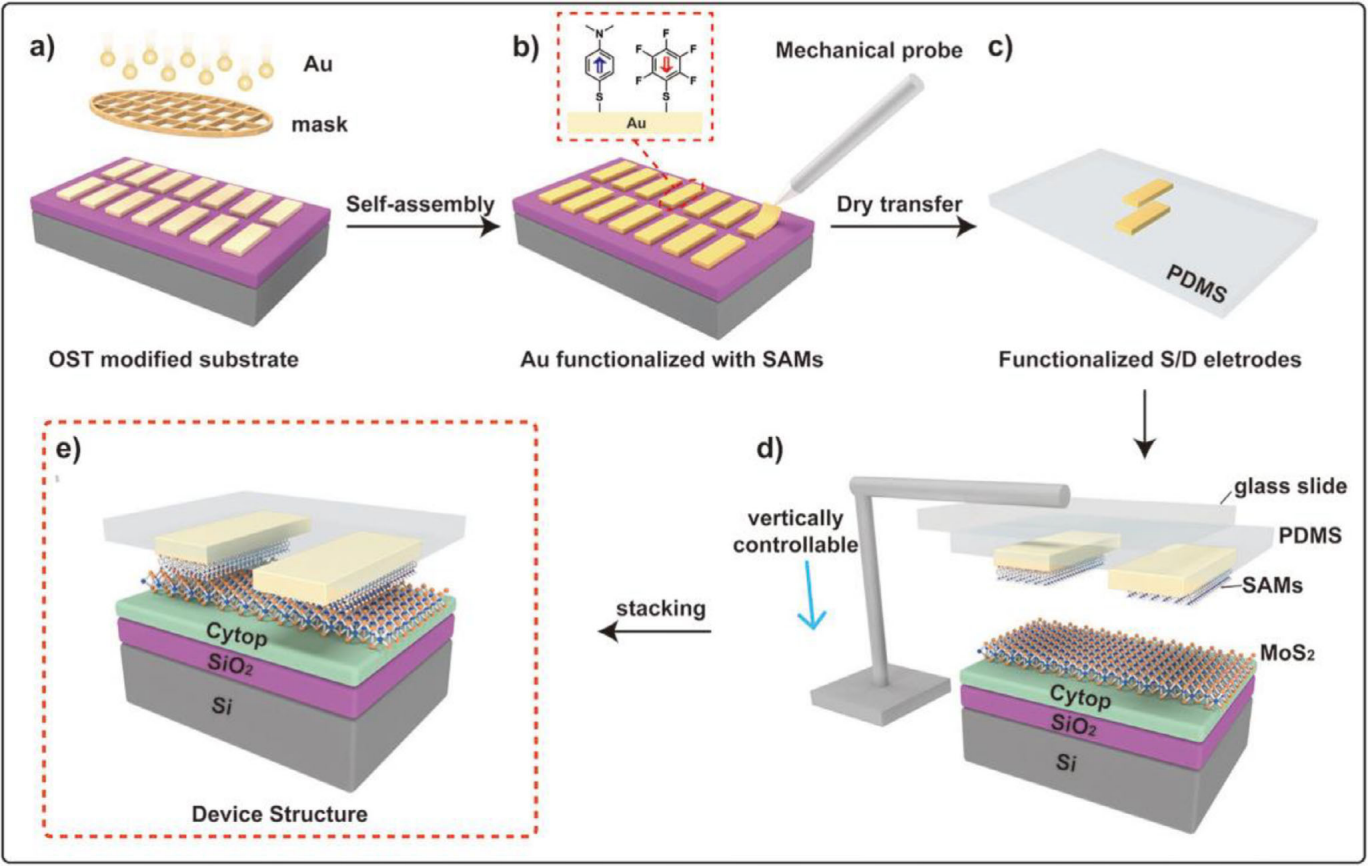
Schematic illustration of the procedural steps for preparing top‐contact field‐effect transistor (FET) devices with functionalized electrodes. (a) deposition of gold (Au) through a patterned mask onto an octadecyltrichlorosilane (OTS)‐modified substrate, followed by self‐assembly, (b) functionalization of the Au electrodes with self‐assembled monolayers (SAMs) to tailor surface properties, (c) preparation of functionalized source/drain (S/D) electrodes on a polydimethylsiloxane (PDMS) substrate via dry transfer, (d) controlled stacking of the SAM‐functionalized electrodes onto the MoS_2_ channel layer using a vertically adjustable setup and (e) final device structure comprising MoS_2_ as the active layer, Si/SiO_2_ as the substrate, and Cytop as the dielectric layer, forming a complete top‐contact FET device. Reproduced with permission [39]. Copyright 2022, Wiley.

A well‐known example of a material that adopts the 2H structure is molybdenum disulfide (MoS_2_). MoS_2_ has gained significant attention for its excellent electronic and optical properties. It is widely used in transistors, which are essential components in modern electronic circuits. Large‐area MoS_2_ can be synthesized using chemical vapor deposition‐like growth techniques [36]. The absence of dangling bonds also allows for the vertical re‐stacking of different 2D materials to create heterostructures without the need for lattice matching [37, 38]. Unlike traditional silicon‐based transistors, MoS_2_‐based transistors can be made extremely thin and flexible, making them suitable for next‐generation flexible and wearable electronics. Figure 6 shows a hysteresis‐free transistor synthesized by using MoS_2_ with low‐k/high‐k bilayer gate of PMMA/P(VDF‐TrFE) [35]. The device shows a very low threshold voltage (V_TH_) of −2.2 V and minimal hysteresis (ΔV_TH_ ≈ 0.1 V), and the interface trap density was reduced to 7.0 × 10^11^ cm^−2^ eV^−1^. The transistor maintained a stable performance under both positive and negative bias stress over 3600 s, and after 101 continuous operation cycles, no significant degradation was observed. Low‐frequency noise measurements confirmed minimal carrier fluctuation, indicating high device reliability and interface quality. Whereas Han et al. [39] developed a new method to chemically functionalize top‐contact gold electrodes in MoS_2_ FETs using self‐assembled monolayers, as shown in Figure 7.

**FIGURE 8:**
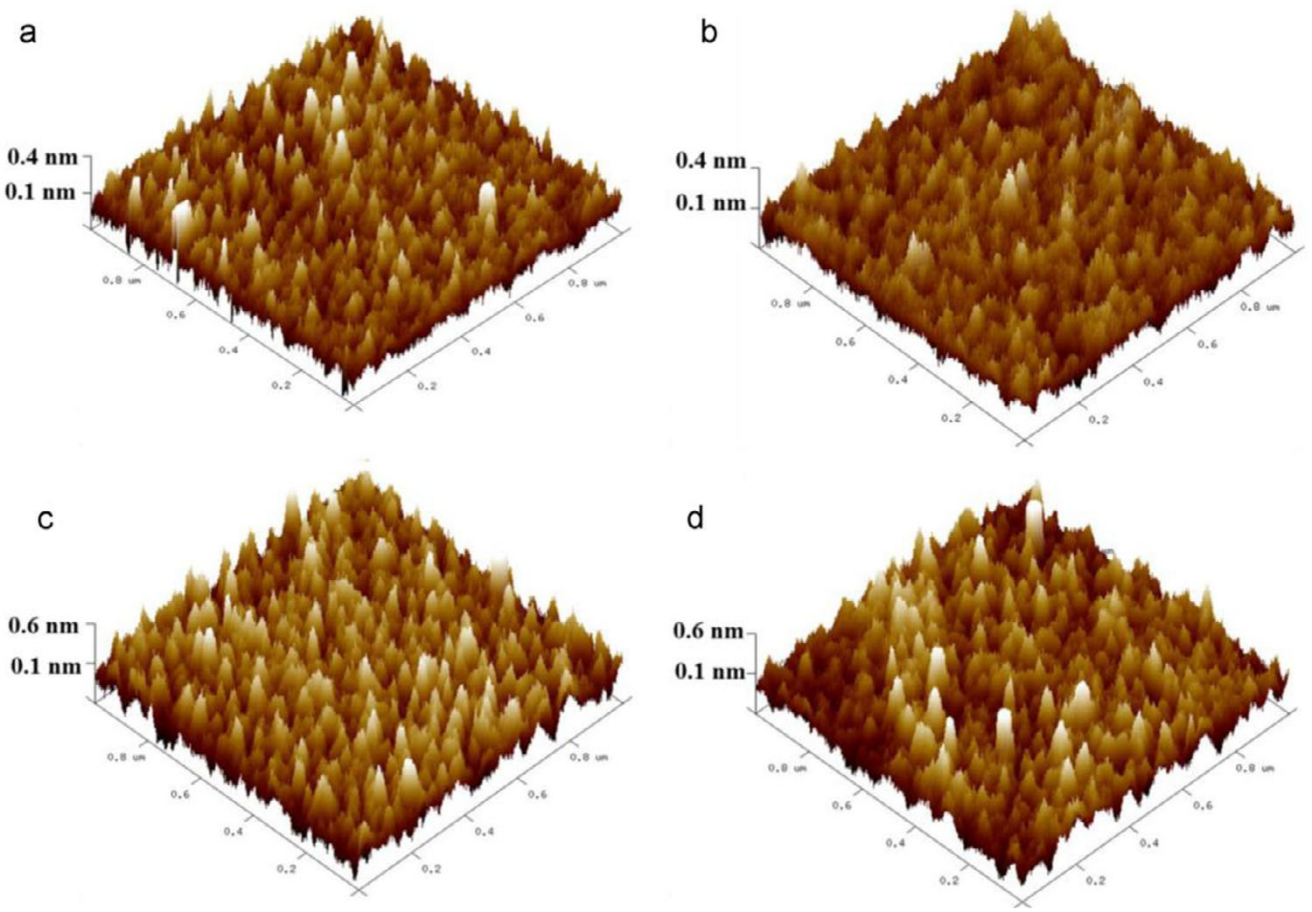
Surface morphology of MoS_2_ films at different substrate temperatures (a) 100°C, (b) 200°C, (c) 400°C and (d) 600°C. Reproduced with permission [41]. Copyright 2016, Elsevier.

By using molecules with different dipole moments, such as DABT (electron‐donating) and PFBT (electron‐withdrawing), they tuned the charge injection barrier and work function of electrodes, which shifted from 5.0 to 4.3 eV with DABT and to 5.67 eV with PFBT. Devices with DABT‐functionalized electrodes achieved a high field‐effect mobility of 30.6 cm^2^/Vs and a low Schottky barrier height (Φ_SB_) of 0.07 eV as compared to that of 0.19 eV for unmodified Au and 0.53 eV for PFBT‐modified electrodes. The study also demonstrated a Schottky diode with a rectification ratio of ∼10^3^ using asymmetric functionalization. This dry‐transfer functionalization technique enables precise control over charge injection and enhances MoS_2_ device performance and reliability. A report has studied a highly sensitive and eco‐friendly MoS_2_‐based FET biosensor functionalized using a DNA tetrahedron structure with a biotin‐streptavidin system for detecting prostate‐specific antigen (PSA). This functionalization method eliminated the need for toxic chemicals like APTES and GA. The biosensor demonstrated an ultralow detection limit of 1 fg/mL and a wide linear detection range from 1 fg/mL to 100 ng/mL in both PBS and undiluted human serum. Real‐time detection showed a rapid response within 2–4 min. The sensor maintained high specificity even in the presence of interfering proteins like IgG and HSA, and had a response variation (%R) of ∼38.6% for 1 fg/mL PSA. The standard deviation in signal response was under 10%, indicating high reliability and repeatability, making this device promising for clinical point‐of‐care applications [40]. Additionally, MoS_2_ is used in photovoltaics, where it helps convert sunlight into electricity in solar panels. Due to its layered structure, MoS_2_ can also be exfoliated into ultra‐thin sheets, creating 2D materials with unique properties. These 2D materials have enhanced electronic and optical characteristics, making them promising for a variety of future applications, including high‐performance sensors, transparent electronics, and even quantum computing. The combination of stability, flexibility, and semiconducting properties makes 2H‐structured materials like MoS_2_ highly valuable in advanced technologies. Solar cells fabricated with p‐Si/MoS_2_ heterojunction using pulsed laser deposition achieved enhanced photovoltaic performance with optimized MoS_2_ film thickness and growth temperature to improve device efficiency [41]. A 12.1 nm MoS_2_ layer grown at 400°C showed the best results, with a short‐circuit current density (J_SC_) of 18 mA/cm^2^, open‐circuit voltage (V_OC_) of 0.39 V, fill factor (FF) of 0.63, and power conversion efficiency (PCE) of 4.5%. Thinner films (<3.8 nm) led to poor light absorption, while thicker films (>21.6 nm) suffered from reduced exciton dissociation. Their surface morphology remained approximately unchanged at different temperatures, as shown in Figure 8. Atomic force microscopy revealed that moderate surface roughness at 400°C enhanced light capture and charge separation. These findings show that thickness and growth conditions critically influence MoS_2_/Si solar cell performance.

The 3R structure is a unique crystal structure that differs from the 2H structure, mainly in how its atomic layers are stacked. While both structures share trigonal prismatic coordination, meaning that each metal atom is surrounded by six chalcogen atoms arranged in a prism‐like shape, the key difference lies in their stacking order. In the 3R structure, the layers follow a rhombohedral symmetry, meaning that each layer is slightly offset from the one below it in a repeating pattern. This contrasts with the 2H structure, where the layers follow a simple hexagonal stacking sequence. The rhombohedral stacking in the 3R structure results in differences in interlayer interactions, which in turn affect the material's electronic and optical properties. One of the most important effects of this structure is its influence on the band gap, which determines how a material interacts with light and electricity. The band gap in 3R materials can differ from that in 2H materials, leading to variations in conductivity and optical absorption. Because of this, materials with a 3R structure often exhibit unique semiconducting or optoelectronic properties, making them useful for applications in electronic devices and energy technologies. A well‐known example of a material that adopts the 3R structure is tungsten diselenide (WSe_2_). This material has gained attention for its electronic and optical properties, which make it suitable for use in photodetectors, solar cells, and other energy‐related applications. Its structure allows it to efficiently absorb and emit light, making it valuable in optoelectronic devices.

**FIGURE 9:**
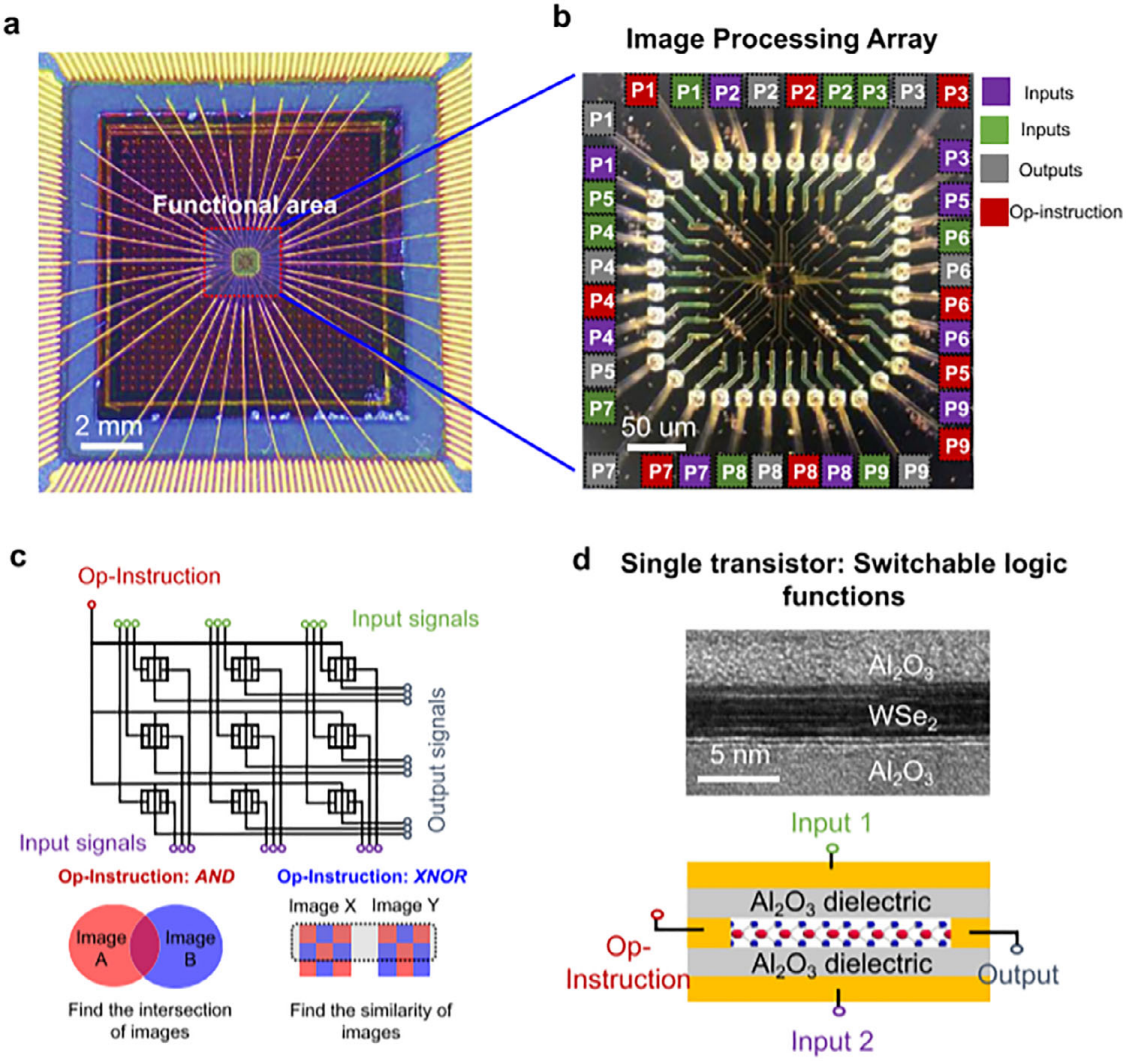
Image processing array with switchable functions. (a) optical image of the fabricated image processing chip showing the functional area (center) where computation occurs. Gold bond wires connect the chip to external circuits for signal input and output, (b) magnified view of the image processing array, highlighting the arrangement of input (green), output (gray), and operation‐instruction (red) terminals. The array enables spatially parallel signal processing for image computation. (c) Schematic illustration of the image processing architecture. Each cell receives input signals (green) and operation instructions (red) to perform pixel‐wise logic operations such as AND (image intersection) or XNOR (image similarity). The outputs correspond to the computed image features, and (d) structure and operation of a single transistor with switchable logic functions. A cross‐sectional TEM image (top) shows the layered Al_2_O_3_/WSe_2_/Al_2_O_3_ heterostructure. Reproduced with permission [42]. Copyright 2022, Nature.

Additionally, WSe_2_ can be exfoliated into thin layers, further expanding its potential for use in next‐generation nanoelectronics and flexible electronics. A WSe_2_/reduced graphene oxide (rGO) nanocomposite synthesized via a hydrothermal process for the oxygen evolution reaction (OER) in water splitting has been reported. The composite exhibited a high surface area (49.83 m^2^/g) and required a low overpotential of 193 mV at 10 mA/cm^2^. It showed a low Tafel slope of 32 mV/dec, indicating fast charge transfer kinetics. The electrochemically active surface area (ECSA) was 500 cm^2^, with an onset potential of 1.35 V vs RHE. Stability testing showed 74.7% capacitance retention over 50 h at 104 mA/cm^2^. The addition of rGO improved conductivity, reduced WSe_2_ agglomeration, and enhanced catalytic performance, making this composite a promising low‐cost and efficient OER catalyst [43]. Other applications of WSe_2_ include a low‐transistor image processing array using WSe_2_ transistors with electrically switchable logic functions (AND/XNOR) by adjusting the drain voltage, as shown in Figure 9. A single WSe_2_ transistor can act as a complete pixel processing unit without the need for extra logic circuits or multiplexers. This results in a dramatic reduction in transistor count (16% of traditional CMOS logic would require). A 3 × 3 transistor array successfully demonstrated basic image processing tasks like finding image intersections (AND) and similarities (XNOR). The design showcased compactness, functional flexibility, and the potential for hardware‐efficient parallel image processing systems [42].

### 2.2 ETM Sesquichalcogenides

The crystal structures of ETM sesquisulfides, such as Ti_2_S_3_, Nb_2_S_3_, Ta_2_S_3_, and Cr_2_S_3,_ are unique and interesting. These materials are often referred to as sesquisulfides because they contain two metal atoms (M) for every three sulfur atoms (S). One of the special features of these compounds is that they can show what is known as “self‐intercalation.” This happens when some metal atoms of the same kind are inserted between already existing layers of metal‐sulfur (MS_2_) sheets. These inserted metal atoms are not part of the regular repeating layer, but they fit in the spaces between the layers. Since not all of these spaces are filled, we call the layers “partially filled.” This kind of structural behavior leads to a chemical formula like M_0.33_MS_2_, which is another way to express M_2_S_3_. For example, in titanium sesquisulfide (Ti_2_S_3_), we can imagine layers of TiS_2_, where some titanium atoms are inserted in between the layers. These extra titanium atoms help to stabilize the structure, even though they don't fill every space between the layers. A similar behavior is seen in niobium sesquisulfide (Nb_2_S_3_), tantalum sesquisulfide (Ta_2_S_3_), and chromium sesquisulfide (Cr_2_S_3_). These metals also insert some of their atoms between MS_2_ layers in a way that affects the overall structure and properties of the material.

**FIGURE 10:**
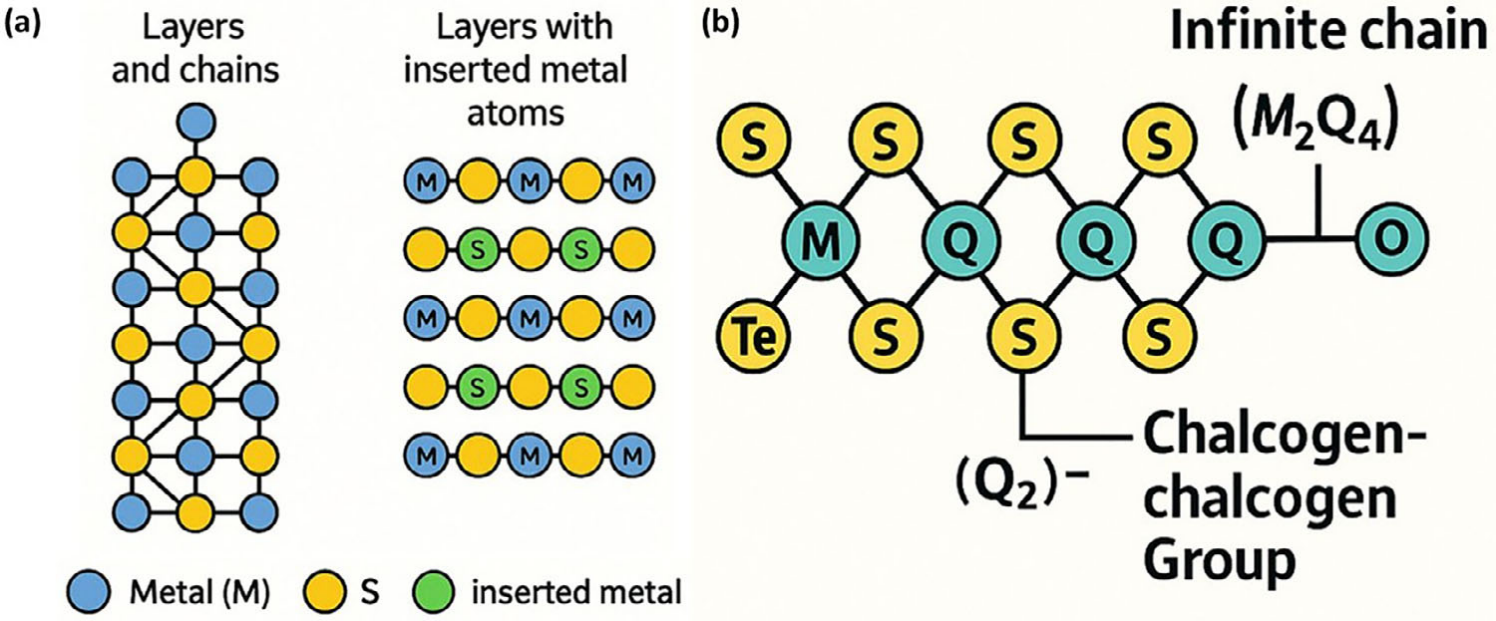
(a) Atomic structure showing the layered and chain‐like arrangement of M_2_Q_3_ compounds, composed of alternating layers of metal (M, blue) and chalcogen (S, yellow) atoms. The metal atoms form interconnected chains within the layered framework (on left), and a modified structure showing layers with inserted metal atoms (green), where additional metal atoms are incorporated between the existing M‐S layers (on right) and (b) schematic illustration of the atomic structure of transition metal trichalcogenides (M_2_Q_3_) consisting of infinite chains of linked (M_2_Q_4_) units, where transition metal atoms (M, cyan) are coordinated by chalcogen atoms (Q, yellow). The chains are interconnected by chalcogen‐chalcogen Q_2_^−^ groups, which stabilize the crystal framework. The presence of terminal atoms such as tellurium (Te) and oxygen (O) demonstrates possible variations in chain termination and bonding environments.

Another important feature of these sesquisulfides is that they can appear in different forms known as polymorphs. Polymorphs are different structural versions of the same compound. Even though the chemical formula is the same, the way the atoms are arranged in space can change. In the case of M_2_S_3_ compounds, the layers of MS_2_ can be stacked in various ways. Sometimes, the stacking is simple and repeats in a regular pattern, but in other cases, the stacking can be more complex, involving multiple layers arranged in different sequences, leading to different crystal structures and physical properties. For instance, some polymorphs of Cr_2_S_3_ can show chain‐like or ribbon‐like arrangements of atoms rather than just flat sheets. In other cases, materials like Nb_2_S_3_ might display more compact, 3D‐like arrangements due to the way the metal atoms are positioned between the sulfur layers. Figure 10a illustrates different stacking styles of M_2_Q_3_ compounds. “Q” here stands for chalcogen, and in these examples, sulfur is the chalcogen. The figure would help visualize how different M_2_S_3_ compounds are built from layers and chains and how inserting metal atoms in between affects the structure. It's significant to highlight the pronounced disparities in the magnetic and electronic characteristics of these compounds, which arise from the varying electronic configurations of the metal ions.

For instance, Cr_2_S_3_ (Cr^3+^, d^3^) behaves as a ferrimagnet and displays semiconductor properties, whereas Ti_2_S_3_ (Ti^3+^, d^1^) is diamagnetic and demonstrates metallic conductivity [44]. It's important to mention that the self‐intercalation structure is not the only exclusive feature of M_2_Q_3_ phases. For instance, in molybdenum sesquisulfide (Mo_2_S_3_), Mo adopts a distinctive structure characterized by metal chains in a zig‐zag fashion of two distinct sorts formed within its layers, featuring Mo–Mo distances of approximately 2.86 Å. In the Mo_2_S_3_ structure, Mo atoms are positioned within the distorted hexagonal octahedra of S atoms, with the unique layers being connected by covalent Mo‐S bonds [45]. Similarly, niobium and tantalum sesquiselenides, Nb_2_Se_3_ (Nb^3+^) and Ta_2_Se_3_ (Ta^3+^), both with d^2^ configuration, respectively, adopt this modification at elevated temperatures [46].

### 2.3 ETM Polychalcogenides

Among the polychalcogenides of early transition metals, a lot of research has been done on a special group of compounds called trichalcogenides (Figure 10b). These are generally written as MQ_3_, where M stands for metals from Group IV or Group V in the periodic table‐such as titanium (Ti), tantalum (Ta), zirconium (Zr), niobium (Nb), and hafnium (Hf) and Q represents a chalcogen element like sulfur (S), selenium (Se), or tellurium (Te) [47]. These MQ_3_ compounds have drawn interest because of their unique structures and the interesting physical and electronic properties they show, which make them promising for advanced materials and devices. A particularly important feature of these trichalcogenides is the presence of long, repeating chains made up of structural units called {M_2_Q_4_} fragments. These fragments are connected to form infinite chains that run throughout the crystal. Some well‐known examples of this type of compound include VS_4_, NbS_4_, V_2_Se_9_, Nb_2_Se_9_, NbTe_4_, and TaTe_4_. These materials often contain special chalcogen‐chalcogen groups known as (Q_2_)^2−^ units, which are pairs of chalcogen atoms bonded together within the structure. These (Q_2_)^2−^ units carry a negative charge and play a key role in the structure and chemistry of these compounds.

For the group IV metals, specifically titanium (Ti), zirconium (Zr), and hafnium (Hf), a series of compounds has been reported in the literature that also feature long chains. These chains often carry a negative charge and are more complex in structure. A good example to understand their general design is zirconium triselenide (ZrSe_3_), which is often used as a model or reference compound. In ZrSe_3_, the basic structural building block is a chain of {ZrSe_6_} trigonal prisms. These prisms are connected to each other by sharing their triangular faces, forming long columns. The shape of each prism's base is not a perfect triangle but rather an isosceles triangle. This happens because there are selenium‐selenium (Q‐Q) bonds inside the structure, where two selenium atoms are covalently bonded. This internal bonding is important, and it is reflected in the compound's chemical formula [48]. The formula for these trichalcogenides is often written as M^4+^Q^2−^(Q_2_)^2−^ to show the presence of both individual chalcogen atoms and bonded chalcogen pairs. One interesting thing about these MQ_3_ structures is that they can have different levels of symmetry and complexity. Such as in niobium trisulfide (NbS_3_), there is only one type of trigonal prismatic column. In tantalum triselenide (TaSe_3_), two different types of columns are found, and in compounds like tantalum trisulfide (TaS_3_) and niobium triselenide (NbSe_3_), there are three different types of prismatic columns. These structural differences lead to variations in physical properties, such as electrical conductivity or how the material behaves under different temperatures or pressures. In addition to trichalcogenides, Group V metals such as vanadium (V), niobium (Nb), and tantalum (Ta) also form compounds called tetrachalcogenides, written as MQ_4_. In these compounds, each chalcogen atom (Q) has an oxidation state of ‐1 [49, 50]. A good example is vanadium tetrasulfide (VS_4_), which occurs naturally as the mineral patronite [51]. In the structure of VS_4_, vanadium atoms with a +4 charge (V^4+^) pair up to form V_2_ units. These pairs are connected by metal‐metal bonds, known as d^1^‐d^1^ bonds, meaning each vanadium atom contributes one electron to the bond, giving the structure added stability [52]. Niobium tetrasulfide (NbS_4_) is structurally very similar to VS_4_ and shares many of the same features.

### 2.4 Lower ETM and Other Chalcogenides

Some early transition metals, such as niobium (Nb) and molybdenum (Mo), can form special types of compounds with chalcogen elements like sulfur (S), selenium (Se), and tellurium (Te). These compounds are called lower chalcogenides because they contain fewer chalcogen atoms in their chemical formula compared to other common chalcogenides. Two important examples of these lower chalcogenides are those with the formulas Nb_3_Q_4_ and Mo_3_Q_4_, where Q can be sulfur, selenium, or tellurium. Even though these formulas might look similar, the actual structures of these materials are quite different and interesting. In the case of niobium‐based chalcogenides, such as Nb_3_S_4_, Nb_3_Se_4_, or Nb_3_Te_4_, the crystal structure is built from units called {NbQ_6_} octahedra. These are shapes where one niobium atom is surrounded by six chalcogen atoms in an octahedral arrangement. However, these octahedra are not perfectly shaped‐they are distorted, meaning the bond lengths and angles are slightly irregular. These distorted octahedra connect by sharing both faces and edges, which is a bit unusual in crystal chemistry. Because of this connection pattern, a fascinating structure is formed: zigzag chains of metal atoms, specifically –Nb–Nb–Nb–, that run in one particular direction within the crystal. These chains are sometimes referred to as “metal‐metal bonded chains” because the niobium atoms are closely connected, suggesting possible metallic bonding or electron sharing between them [53, 54]. These chains play an important role in giving the material its electrical and structural properties such as Nb_3_Se_4_ is known to have interesting electronic behavior and can even show metallic conductivity.

Alternatively, molybdenum‐based compounds with the formula Mo_3_Q_4_ (where Q is Se or Te) show a completely different kind of structure, even though their composition appears similar at first glance. In reality, the formula for these molybdenum compounds needs to be doubled to reflect their actual structure. That means the true structural formula is something like Mo_6_Q_8_. This is because their crystal structure includes large clusters of atoms arranged in the form of M_6_Q_8_ octahedra, where six molybdenum atoms are bonded together in a cluster and are surrounded by eight chalcogen atoms. These clusters form the basic building blocks of the material, and they repeat in a regular pattern throughout the crystal. These types of cluster‐based structures are quite different from the chain‐like structures found in the niobium chalcogenides [55, 56]. Mo_6_Te_8_ is one such compound with this kind of cluster structure. It shows properties that are influenced by the unique bonding between the molybdenum atoms inside the clusters. These clusters help stabilize the structure and can lead to interesting optical, electronic, and even magnetic behaviors, depending on the exact composition and how the atoms are arranged. Some ETM‐based chalcogenides form 3D crystal structures instead of the more common layered structures. These 3D structures have strong chemical bonds extending in all directions, making them more rigid and stable. Two well‐known types of 3D structures in ETM‐based chalcogenides are the pyrite‐type and spinel‐type structures. These structures differ in how metal and chalcogen atoms are arranged, leading to variations in their properties and applications. The pyrite‐type structure is one of the most recognized 3D frameworks found in ETM‐based chalcogenides. In this structure, the metal atoms are octahedrally coordinated, meaning that each metal atom is surrounded by six chalcogen atoms arranged in an octahedral shape. The chalcogen atoms also form strong covalent bonds with each other, creating a robust and interconnected framework. This unique arrangement results in high electrical conductivity, as the electrons can move easily through the material. It also enhances the material's catalytic activity, making it useful in chemical reactions that require electron transfer. A well‐known example of a material that adopts the pyrite‐type structure is iron disulfide (FeS_2_), commonly referred to as pyrite or “fool's gold” due to its metallic luster and golden color. Pyrite is an abundant mineral found in nature and has been extensively studied for its electronic and optical properties. Because of its high electrical conductivity and ability to absorb sunlight efficiently, FeS_2_ is being explored for use in solar cells as a low‐cost, earth‐abundant alternative to traditional semiconductor materials. Its ability to store and transfer energy also makes it a promising candidate for energy storage devices, such as rechargeable batteries and supercapacitors. In addition to its applications in solar energy and energy storage, pyrite‐type FeS_2_ is also investigated for its role in catalysis. Its ability to facilitate chemical reactions, especially in processes like hydrogen evolution and carbon dioxide reduction, makes it a valuable material in the field of sustainable energy. Investigations have been done on improving its stability and optimizing its properties for use in industrial and environmental applications.

**FIGURE 11:**
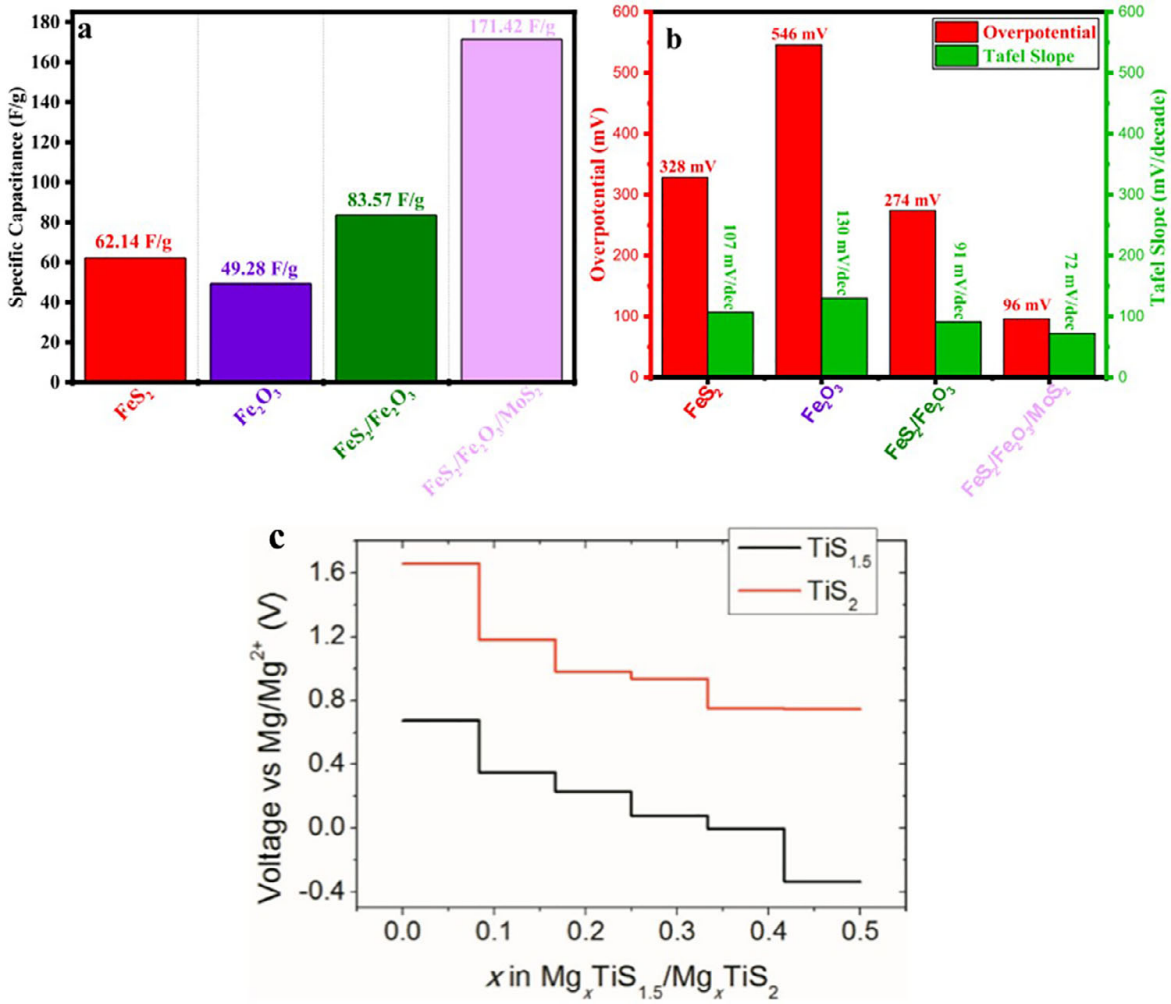
(a) Specific capacitance values illustrating the enhanced charge‐storage capability achieved through material hybridization, with the FeS_2_/Fe_2_O_3_/MoS_2_ composite exhibiting the highest capacitance (171.42 F/g), (b) overpotential and Tafel slope measurements highlighting the electrocatalytic behavior of the samples. The FeS_2_/Fe_2_O_3_/MoS_2_ composite shows significantly improved catalytic activity, reflected in its lower overpotential (96 mV) and reduced Tafel slope, indicating faster reaction kinetics compared to the individual and binary components. Reproduced with permission [57]. Copyright 2025, Elsevier and (c) calculated voltage profiles for TiS_1.5_ and TiS_2_ during Mg insertion. The plots show the stepwise variation in voltage as a function of Mg content (x) in Mg_x_TiS_1.5_ and Mg_x_TiS_2_. TiS_2_ exhibits consistently higher voltages, indicating stronger Mg‐host interactions compared to TiS_1.5_. The plateaus correspond to distinct two‐phase regions formed during magnesiation, providing insights into the thermodynamically stable intermediate phases and the relative electrochemical behavior of the two sulfide hosts. Reproduced with permission [59]. Copyright 2019, Elsevier.

By controlling the synthesis conditions, doping with other elements, or altering the particle size, researchers aim to develop pyrite‐based materials with improved efficiency and durability, paving the way for their use in next‐generation energy and electronic devices. A ternary composite of FeS_2_, Fe_2_O_3_, and MoS_2_ was synthesized via hydrothermal methods, demonstrating excellent dual‐function performance in hydrogen evolution reaction (HER) and supercapacitors [57]. The FeS_2_/Fe_2_O_3_/MoS_2_ showed the best HER activity with a low overpotential of 96 mV at 10 mA/cm^2^, a Tafel slope of 72 mV/dec, and a high electrochemical surface area (ECSA) of 27.79 µF/cm^2^. It also exhibited the lowest charge transfer resistance (R_CT_ = 288 Ω). As a supercapacitor, the composite delivered a high specific capacitance of 171.42 F/g at 1 A/g (Figure 11a,b). The synergy among the three components improved charge transfer and catalytic efficiency, making it suitable for energy and storage applications. A flexible FeS_2_‐based all‐solid‐state lithium battery (ASSLB) was synthesized using a PTFE‐based dry‐process method that operates under a low stacking pressure of 100 MPa, five times lower than traditional methods. The FeS_2_@C/rGO composite cathode and Li_6_PS_5_Cl solid electrolyte formed thin membranes with strong interfacial adhesion. The batteries demonstrated excellent electrochemical performance with a stable discharge capacity of 370.7 mAh/g at 0.3C after 200 cycles. The PTFE binder helped retain electrochemically sluggish products (like Fe and Li_2_S) and suppressed capacity fading. This method also enabled large‐scale, flexible pouch‐cell production, showing promise for industrial applications [58].

The spinel‐type structure is a special type of crystal arrangement where metal and chalcogen atoms form a cubic pattern. This structure is similar to that found in spinel oxides, which are widely known for their stability and unique electrical properties. In a spinel structure, metal atoms occupy specific positions within the cubic framework, while chalcogen atoms surround them in a well‐organized manner. This structural arrangement results in strong atomic bonding, which gives the material excellent mechanical and chemical stability. Because of this, spinel‐type materials can withstand high temperatures, resist degradation over time, and maintain their structural integrity under various conditions. One of the most important characteristics of spinel‐type chalcogenides is their ability to conduct both electrons and ions efficiently. This property, known as mixed electronic‐ionic conductivity, makes these materials particularly valuable in energy storage applications. In a battery, ions move between the electrodes during charging and discharging, while electrons flow through an external circuit to generate electricity. Materials with high ionic and electronic conductivity can improve battery performance by allowing faster charge transfer and reducing energy loss. The spinel structure naturally provides pathways for both ionic and electronic movement, which enhances the overall efficiency of devices that use these materials.

Titanium‐based sulfide spinels, such as Ti_2_S_3_, are among the most well‐researched materials that exhibit this structure. These compounds have drawn significant interest due to their ability to store and release large amounts of energy. When used in batteries, Ti_2_S_3_ can hold a high charge capacity, meaning it can store more energy compared to other conventional materials. This is particularly important for applications that require long‐lasting power sources, such as electric vehicles and renewable energy storage systems. Another advantage of Ti_2_S_3_ and similar spinel sulfides is their long cycle life. Batteries made from these materials can undergo many charge and discharge cycles without significant loss of performance. This durability makes them ideal for applications where reliability and longevity are essential. Also, their ability to conduct both electrons and ions efficiently opens possibilities for use in fuel cells, supercapacitors, and other energy‐related applications. By introducing different elements into the spinel framework, researchers aim to optimize conductivity, stability, and overall performance. As a result, spinel‐type chalcogenides continue to be an exciting area of research, with the potential to revolutionize energy storage and other critical technologies. A study used computational methods to explore Ti_2_S_3_ (TiS_1.5_) as a new cathode for magnesium (Mg) batteries and compared it with well‐known TiS_2_ results. Ti_2_S_3_ showed a high theoretical capacity of 112 mAh/g, an excellent electronic conductivity with zero band gap, and moderate stability, but a lower voltage (by 0.8–1.0 V) due to a weaker redox couple (Ti^3+^/Ti^2+^). Despite an 8.17% contraction in the c‐lattice parameter, Ti_2_S_3_ maintained similar Mg migration barriers (1.078–1.281 eV) as TiS_2_. This is attributed to a lower partial Ti charge and softer bonding, which improved the Mg mobility [59]. Figure 11c shows the electrode potentials vs. the Mg content, which were calculated by using static density functional theory at 0 K temperature. In addition to the commonly observed crystal structures, ETM‐based chalcogenides can also form less common structural arrangements depending on the combination of metal and chalcogen elements. These structures can vary due to differences in atomic size, electronic configurations, and external factors such as temperature and pressure. Some of these less common crystal structures include monoclinic and orthorhombic phases, which differ in their atomic arrangements and symmetry. The monoclinic crystal structure is characterized by a unit cell where the three axes have different lengths, and only one of them is perpendicular to the others. This leads to a structure that lacks the high symmetry of hexagonal or cubic systems but can offer unique electronic and mechanical properties [8, 13]. Some ETM‐based chalcogenides crystallize in this form when the interactions between the metal and chalcogen atoms result in slightly distorted bonding angles. The distortion in atomic arrangements can cause variations in the material's electrical conductivity and optical behavior, making them useful for specialized applications. These materials can also transition between different crystal phases depending on external conditions, such as pressure or temperature changes. The orthorhombic crystal structure, on the other hand, has three axes of different lengths, all of which remain perpendicular to one another. This structure is more ordered than the monoclinic phase but still differs from more symmetric systems like cubic or hexagonal arrangements [13, 60]. Some ETM‐based chalcogenides can crystallize in the orthorhombic phase when their atomic arrangements create anisotropic properties, meaning the material behaves differently along different directions. This can be beneficial for applications requiring directional conductivity or controlled mechanical properties.

## 3 Chemical and Structural Aspects of ETM‐Based Chalcogenides

Chalcogenides are compounds made of a transition metal and two chalcogen atoms are very useful starting materials for creating other important chemical compounds. The specific behavior and chemical properties of these chalcogenides largely depend on the type of transition metal and chalcogen they contain [61, 62]. One example is the group of compounds known as molybdenum chalcogenides, such as molybdenum disulfide (MoS_2_), molybdenum diselenide (MoSe_2_), and molybdenum ditelluride (MoTe_2_). When these compounds are heated to high temperatures and begin to break down (a process called thermolysis), the resulting products vary depending on which chalcogen (S, Se, or Te) is involved. If molybdenum disulfide (MoS_2_) is thermally decomposed, it forms Mo_2_S_3_, a compound with a different ratio of sulfur and molybdenum. Comparatively, if molybdenum diselenide (MoSe_2_) or molybdenum ditelluride (MoTe_2_) is heated, the products are typically Mo_3_Q_4_ compounds (where Q represents either selenium or tellurium) [63, 64, 65]. These differences highlight how the nature of the chalcogen element influences the chemical transformations and the final structure of the compounds formed.

Rhenium chalcogenides, which contain the metal rhenium (Re), are especially interesting because rhenium has more valence electrons compared to other transition metals in this group [66]. This gives rise to unique and sometimes surprising chemical behavior. For example, when rhenium disulfide (ReS_2_) reacts with chlorine, it doesn't oxidize the rhenium as might be expected. Instead, two chlorine atoms replace one sulfur atom, forming a compound called thiochloride (ReSCl_2_). Further reduction of ReSCl_2_ in hydrogen produces rhenium monosulfide (ReS), which is amorphous, meaning it doesn't have a regular crystal structure, quite unusual for this system [67, 68, 69]. In another case, when rhenium diselenide (ReSe_2_) is reacted with bromine at high temperatures around 800°C, it forms a compound with six rhenium atoms bonded together, known as a hexanuclear cluster, showing even more complex chemical behavior [70], as given below:

(1) $$\mathrm{ReS}e_{2}+Br_{2}\to Re_{6}Se_{4}Br_{10}$$

It's worth noting that this reaction is associated with Re reduction from Re^4+^ to Re^3+^, which may appear somewhat unexpected considering the relatively high oxidizing potential of bromine. Enhancements in application‐specific properties were achieved not solely through the utilization of nanostructured materials but also through the precise adjustment of electronic properties via atom substitutions in both anion and cation sub‐lattices and intercalation [71, 72]. As demonstrated, for example, the partial substitution of Mo atoms in MoS_2_ with Re atoms results in a significant reduction in sample resistance and an increase in tensosensitivity [73]. Similarly, partial substitution with Nb atoms brings about a change in conductivity type from semiconductor to metallic behavior [74]. This process has proven to be highly effective in tailoring thermoelectric properties.

In contrast to layered chalcogenides, low‐dimensional transition metal polychalcogenides exhibit distinctive features such as Van der Waals gaps between infinite layers (e.g., NbSe_3_, TaS_3_) or chains (e.g., VS_4_, V_2_Se_9_) [75]. These gaps create chances for intercalation, stratification, dispersion, and the formation of nanostructures down to individual chains or layers. Unlike MQ_2_ compounds, polychalcogenides contain (Se‐Se)^2–^ or (S‐S)^2–^ groups proficient in undergoing reversible redox alterations. These unique characteristics make polychalcogenides of transition metals a subject of current research interest as electrode materials in energy storage devices, including lithium/sodium‐ion batteries and supercapacitors [76, 77].

Furthermore, the layered or chain configurations found in chalcogenides have the potential to enhance stable cycling by accommodating intercalation processes more effectively. Additionally, the (S‐S)^2–^ fragments play a role in electrocatalytic processes, such as the hydrogen release reaction during water decomposition, which is currently of practical significance and extensively researched [78]. A substantial body of data indicates that the ‐S‐S‐disulfide groups serve as active centers in electro‐ and photocatalysts based on materials like FeS_2_, MoS_3_, and ZrS_3_ [79]. Similar investigations have been conducted for polyselenides as well. Also, it is essential to consider both the metal and ligand influence in conjunction [80]. An intriguing example illustrating the impact of these factors on electronic structures is observed in Mo chalcogenides [81]. Significant structural changes occur when low‐dimensional compounds, such as the transition from the layered structure of MoSe_2_ to the bulk structure of octahedral clusters in Mo_6_Se_8,_ replace chalcogenides [82]. These changes appear to be primarily driven by the electronic characteristics of the elements, particularly the metal atoms, but ligands also play a substantial role. The number of valence electrons in the metal atoms, the sizes, the electronegativity of the chalcogenide ligands, and the consequent compound structures are all delicately balanced in these systems [83, 84]. For instance, lower selenide and telluride compounds adopt the composition Mo_3_Q_4_ in molybdenum‐chalcogen systems, while sesquisulfide Mo_2_S_3_ crystallizes in these systems [85]. This is attributed to the higher electron density of molybdenum atoms, contributed by the less electronegative (compared to sulfide ions) selenide and telluride ligands [86]. Interestingly, the conversion of Mo_3_Se_4_ selenide (with 20 cluster skeletal electrons per Mo_6_ cluster) into stable Chevrel phases like MMo_6_Se_8_ (with 22 cluster skeletal electrons) is relatively straightforward, whereas telluride Chevrel phases such as MMo_6_Te_8_ are less common (e.g., Ni_0.85_Mo_6_Te_8_, M_x_Mo_6_Te_8_ with M = Ag, Cu; *x* = 1, 2). This disparity is believed to result from an excess of electrons in the telluride compounds [86, 87].

The metastable properties of the Mo_3_S_4_ sulfide phase (Mo_6_S_8_) can also be elucidated by electronic factors, particularly an increased ionic character [88]. However, the introduction of electrons from atoms incorporated during the formation of triple sulfides, like PbMo_6_S_8_ (with 22 electrons per Mo_6_ cluster), leads to its stabilization [89]. A particularly interesting feature of some ETM‐based chalcogenides is their ability to exist in multiple polymorphic forms. Polymorphism refers to the ability of a material to adopt more than one crystal structure under different conditions. This means that a single compound can change its atomic arrangement depending on factors like temperature, pressure, or synthesis methods [90, 91]. By carefully controlling synthesis parameters, one can manipulate these crystal structures to design materials with desired properties. Adjusting factors such as temperature, pressure, chemical composition, and synthesis methods allows the engineering of chalcogenide materials for various technological applications. This flexibility in structural tuning is crucial for advancing materials used in electronics, energy storage, and catalysis. The way layers are stacked in ETM‐based chalcogenides has a major impact on their physical properties. In some crystal structures, the layers are strongly bonded to each other through chemical interactions, leading to stable, rigid structures [92, 93, 94].

However, in many ETM‐based chalcogenides, the layers are held together by weak van der Waals forces. These forces are much weaker than typical chemical bonds, making it easy to separate the layers from one another. Because of this, one can easily exfoliate these materials into very thin layers, sometimes even down to a single atomic layer [95, 96]. This property is highly valuable for creating 2D materials, which have unique optical and electronic characteristics not seen in their bulk form. 2D materials derived from ETM‐based chalcogenides are particularly important in modern research because they offer advantages like high flexibility, transparency, and excellent electronic properties.

**FIGURE 12:**
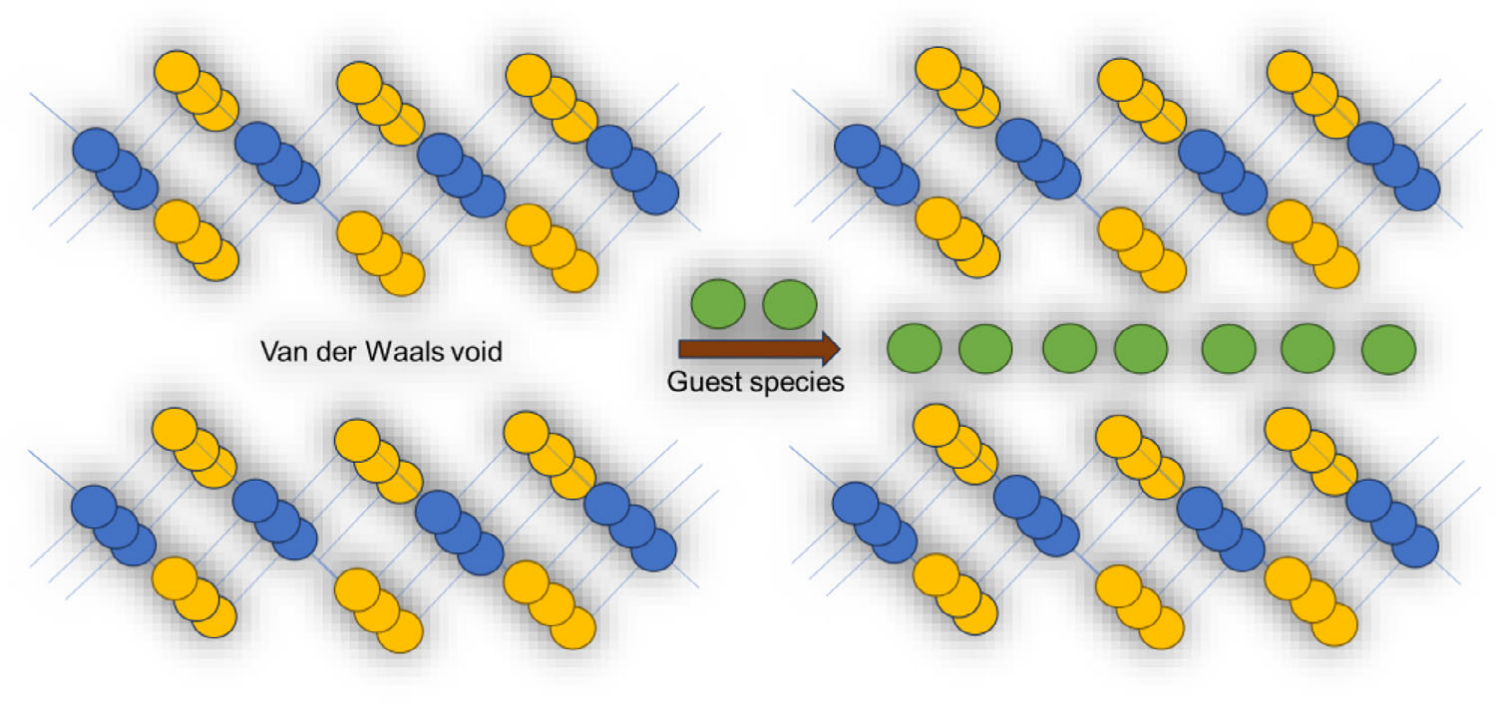
Schematic illustration of the intercalation process in layered ETM‐based chalcogenides. The layered structure, composed of alternating metal (blue) and chalcogen (yellow) atoms, provides van der Waals gaps that allow foreign species (green spheres) to diffuse between adjacent layers. During intercalation, these guest ions or molecules insert themselves into the interlayer spacing, modifying the structural, electronic, and chemical properties of the host ETM‐chalcogenide material.

Many ETM‐based chalcogenides adopt a layered structure, where the TM cations are sandwiched between layers of chalcogen anions, as shown in Figure 12 [97]. The ability to manipulate these layers plays a crucial role in material engineering. Researchers across the globe have developed different methods to modify, rearrange, or chemically alter the stacking of layers to achieve desired properties. One method involves mechanical exfoliation, where adhesive tapes or other mechanical tools are used to peel off thin layers from bulk crystals [98, 99, 100]. Another approach is chemical exfoliation, where specific solutions break the weak van der Waals bonds and separate the layers. These techniques allow the creation of high‐quality, ultra‐thin materials that can be easily integrated into electronic devices. Stacking order can also be deliberately altered to produce new effects. Stacking different types of chalcogenide layers together, creating heterostructures with custom properties, has also been explored [101, 102, 103]. These structures can combine the strengths of multiple materials, leading to improvements in conductivity, light absorption, and charge mobility. By carefully adjusting how layers interact, one can design materials suited for next‐generation transistors, memory devices, and energy storage systems.

Phase‐change memory (PCM) devices made from stacked layers of Ge‐based and Sn‐based chalcogenides were fabricated and tested. The tested structures include GeTe/SnTe, Ge_2_Se_3_/SnTe, and Ge_2_Se_3_/SnSe [104]. All devices demonstrated resistance switching, meaning they could store data by switching between high (amorphous) and low (crystalline) resistance states. It was observed that for GeTe/SnTe devices, switching occurred at threshold voltages below 1.8 V, and multi‐state switching behavior was observed. Devices had initial resistances around 45 MΩ, which reduced to as low as 0.5 kΩ after switching with ±1 mA. For Ge_2_Se_3_/SnTe devices, higher threshold voltages (∼3.7 V) were required, but they showed better device‐to‐device consistency and lower switching current (∼100 µA). This was attributed to better film quality and step coverage using sputtered Ge_2_Se_3_. Ge_2_Se_3_/SnSe devices required a “conditioning” step (applying a small current at 3 V) to trigger phase‐change switching under negative bias, likely due to Sn ion migration into the Ge_2_Se_3_ layer. Without this conditioning, switching did not occur. No switching was observed when only Se ions were likely to move, indicating the importance of Sn or Te ion migration for activation. Layered chalcogenides like Ge_2_Se_3_Te, GaSe, and Sb_2_Te_3_ have also been studied, and how atomic bonding affects stacking defects in these materials, making them useful for data storage and thermoelectrics, has also been explored. A recent study compared the stable hexagonal and metastable rhombohedral phases of Ge_2_Se_3_Te using electron microscopy and X‐ray diffraction. The hexagonal phase exhibited many planar defects called translational shear faults (TSFs), caused by weak van der Waals (vdW) bonding between layers. The TSFs were confirmed by broadened (*h*0*l*) peaks in XRD and were most prominent when a 50% fault probability was modeled. The rhombohedral phase showed no such faults. Similarly, GaSe also showed TSFs with a 50% occurrence, indicating weak interlayer interactions. In contrast, Sb_2_Te_3_ exhibited no stacking defects and had much stronger bonding across its layers. Its Te‐Te interlayer distance was 3.711 Å, which is 0.45 Å shorter than the vdW distance (4.16 Å), suggesting a bonding type stronger than vdW, possibly metavalent bonding. The DFT study confirmed that stacking defects in GaSe cost very little energy to form, while in Sb_2_Te_3_, defects significantly changed energy, bandgap (increased from ∼0.0 to 43 meV), and dielectric properties. These findings suggest that while GaSe and Ge_4_Se_3_Te behave like 2D vdW materials, Sb_2_Te_3_ has 3D‐like bonding and should not be labeled as purely 2D. This study highlights how bonding strength directly affects defect formation and material performance [105]. So, it can be said that the stacking order of ETM‐based chalcogenides plays a vital role in defining their properties.

In addition to their structural diversity, ETM‐based chalcogenides also exhibit a range of phase transitions when exposed to external factors such as temperature, pressure, or electric fields. These transitions involve changes in the arrangement of atoms within the crystal lattice, which can significantly affect the material's electronic, optical, and mechanical properties. Some materials can shift from one crystal structure to another depending on environmental conditions, leading to variations in their conductivity, magnetism, or stability. One notable example is the transition from a 2H phase to a 1T phase in certain ETM‐based chalcogenides [105, 106, 107]. The 2H phase has trigonal prismatic coordination, which generally results in semiconducting behavior, while the 1T phase has an octahedral coordination that often exhibits metallic properties. This transformation can be induced by applying an external voltage, changing the temperature, or introducing chemical modifications, allowing us to manipulate material properties for specific applications.

**FIGURE 13:**
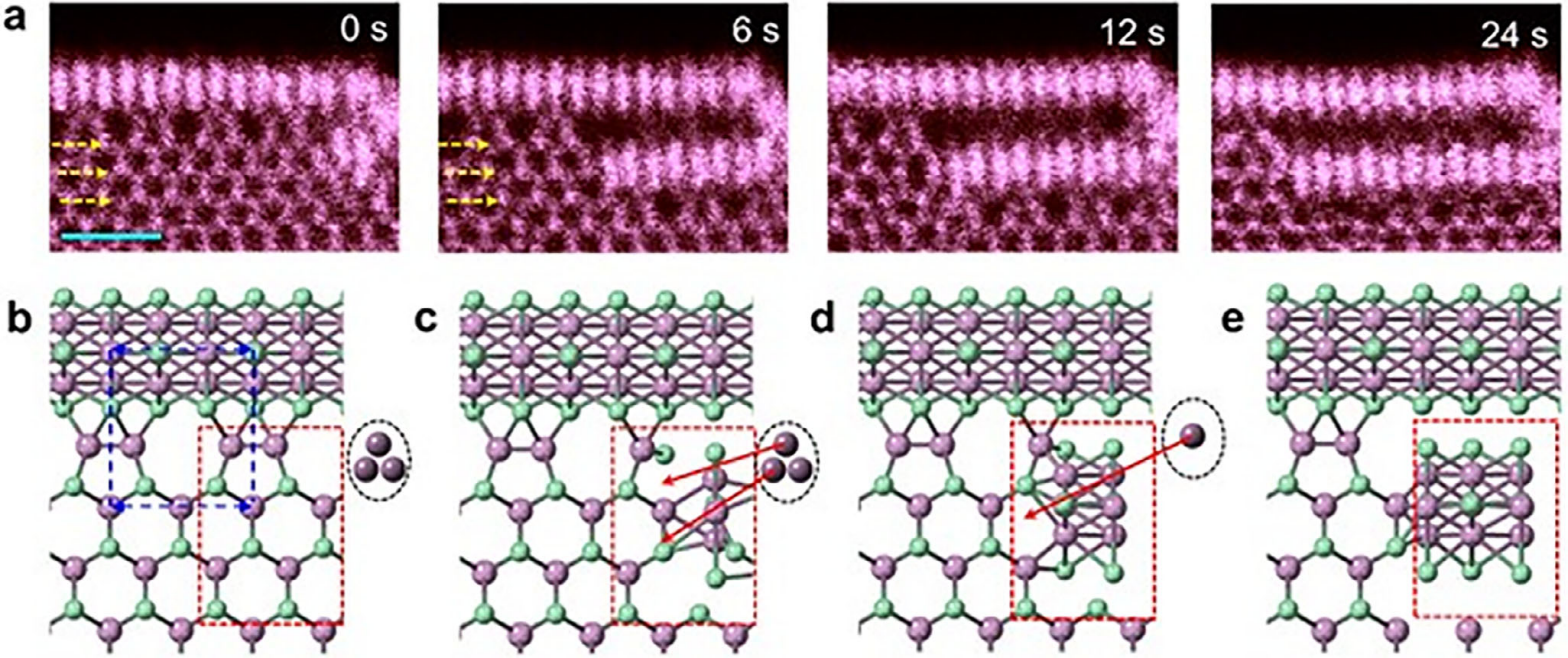
Atomic mechanism for MoSe_2_ → Mo_6_Se_6_. a) Experimental dynamic process of MoSe_2_ → Mo_6_Se_6_. Scale bar: 1 nm. The three rows of Se_2_ sublattice marked by yellow arrows signify the vertical unit size of the MoSe_2_ → Mo_6_Se_6_ process. b‐e) Atomic process of MoSe_2_ → Mo_6_Se_6_ within the supercell unit marked by the dashed red rectangle. In this supercell, 6 Mo and 12 Se atoms gradually reconstruct into three Mo_3_Se_3_ rings in e with an extra supply of 3 free Mo atoms (dashed black ellipse). Reproduced with permission [107]. Copyright 2022, Wiley.

A recent paper [107] studied the new 2D phases formed in monolayers of MoS_2_ and MoSe_2_, two popular transition metal dichalcogenides, using in situ scanning transmission electron microscopy. These materials were exposed to electron beams and heated up to 550°C–750°C, which triggered chalcogen‐deficient transformations into novel structures like Mo_4_S_6_, Mo_6_Se_6_, and an intermediate Mo_3_S_4_ phase. The team observed atomic‐scale transitions (as shown in Figure 13):

(2) $$\mathrm{Mo}S_{2}\to Mo_{3}S_{4}-\mathrm{DLV}\to Mo_{4}S_{6}$$

(3) $$\mathrm{MoS}e_{2}\to Mo_{6}Se_{6}\left(Z\;\mathrm{and}\;L\;\mathrm{forms}\right)$$

These changes occurred with atomically sharp boundaries and involved mechanisms like 2H ↔ 1T sliding of atoms and anion vacancies. The energy barrier for Mo_3_S_4_ → Mo_4_S_6_ was calculated to be 0.96 eV, making it thermally accessible above 500°C. The electronic bandgap also changed dramatically as the MoS_2_ direct bandgap (∼2.21 eV) reduced to 0.86 eV in Mo_4_S_6_ and became metallic in Mo_6_Se_6_, enabling semiconductor‐to‐metal transitions. Also, MoS_2_‐Mo_4_S_6_ and MoSe_2_‐Mo_6_Se_6_ heterostructures were synthesized by the group with Schottky barriers as low as 0.24 eV for semiconducting‐semiconducting interface and 0.82 eV for metal‐semiconductor interface. The newly discovered phases were reported to have different work functions and structural stability, which was confirmed by DFT calculations. Phase transitions in ETM‐based chalcogenides play an important role in modern technology, particularly in memory storage devices and electronics. The ability of these materials to switch between different structural phases with distinct electronic properties makes them suitable for use in phase‐change memory devices. In these devices, data is stored by rapidly switching between phases with different resistances, enabling fast and efficient information processing.

The optical response of Ge_2_Sb_2_Te_5_ (GST) phase‐change material can be reversibly tuned from dielectric to plasmonic by switching its structure between the amorphous and crystalline states [106]. The study focused on how these changes affects the color, reflectivity, and light transmission in nano‐patterned thin films, called metasurfaces. GST behaved like a normal dielectric when it was in the amorphous state (positive real permittivity (ε)). However, once crystallized, its ε becomes negative below 660 nm, allowing it to support surface plasmon polaritons. They measured transmission of amorphous GST to increase from ∼5% (at 400 nm) to ∼35% (at 900 nm). In the crystalline state, transmission dropped significantly below 12%. Using nanograting metasurfaces made from a 70 nm GST layer sandwiched between ZnS/SiO_2_, they demonstrated vivid, tunable colors in both transmission and reflection due to resonance effects. The switching contrast in reflection reached above 50%, and the color change was mapped using CIE 1931 color coordinates, showing clear shifts between the two states. The transition was achieved using ultrafast 85‐fs laser pulses at 730 nm. Additionally, some ETM‐based chalcogenides exhibit reversible phase transitions, meaning that they can return to their original structure after the external stimulus is removed. This property is particularly useful in reconfigurable electronics, where materials need to switch between conducting and insulating states without permanent changes to their structure.

Another factor that influences the crystal structure and properties of ETM‐based chalcogenides is the presence of defects and dopants. Defects refer to imperfections within the crystal lattice, such as missing atoms, displaced atoms, or additional atoms that disrupt the regular arrangement of the structure [108, 109]. These defects can have both positive and negative effects on the material's properties. In some cases, defects can create localized states within the electronic band structure, affecting the way electrons move through the material. This can lead to changes in electrical conductivity, optical absorption, and mechanical strength. In other cases, defects can introduce structural instability, reducing the overall performance and durability of the material. A study on how structural defects enable resistive switching in chalcogenide‐based memory devices has been done, which is useful for non‐volatile memory and logic circuits [110]. Three main switching mechanisms were studied, i.e., Phase Change Mechanism (PCM), Thermochemical Mechanism (TCM), and Valence Change Mechanism (VCM). It was observed that in PCM materials (GeSbTe), a short, strong pulse melts the crystalline region, and rapid cooling forms a high‐resistance amorphous state. Recrystallization by a longer pulse switches it back to a low‐resistance state. The resistance difference can be as high as 5 orders of magnitude (∼10^5^×). Crystallization can occur in tens of nanoseconds, and the state remains stable for over 10 years, resolving the voltage‐time dilemma using temperature‐sensitive viscosity models. It was summarized that in TCM, conductive filaments form due to thermal redox reactions; switching is unipolar. Also, VCM involves oxygen ion migration and local redox reactions, resulting in bipolar switching, where different voltage polarities set/reset the device.

**FIGURE 14:**
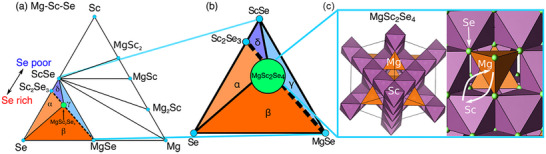
(a) Ternary Mg‐Sc‐Se phase diagram at 0 K computed from DFT data combined with Materials Project data, with (b) a zoom‐in of the concentration range of interest, (c) crystal structure of a normal spinel, such as MgSc_2_Se_4,_ identified in the phase diagrams of panels (a) and (b). The right fragment in panel (c) shows the scenario of spinel inversion (white arrows Mg ↔ Sc) in MgSc_2_Se_4_, leading to antisite MgSc and ScMg defects. Reproduced with permission [111]. Copyright 2017, ACS.

The defect chemistry in spinel chalcogenides like MgSc_2_Se_4_, MgIn_2_S_4_, and MgSc_2_S_4_ was studied, which are promising as solid electrolytes for Mg‐ion batteries (Figure 14) [111]. Although they showed good magnesium ion conductivity (up to 0.1 mS/cm at 298 K for MgSc_2_Se_4_), it was also observed that unwanted electronic conductivity can lead to battery self‐discharge. Using first‐principles calculations, it was found that Mg vacancies and antisite defects (like Sc replacing Mg, denoted by Sc^•^_Mg_) are the main reasons for electronic conductivity. Under anion‐poor synthesis conditions, these defects are more likely to form, increasing n‐type conductivity. Conversely, anion‐rich conditions and slow cooling can reduce defect formation and lower the electronic leakage. The calculated electronic conductivity of MgSc_2_Se_4_ was about 0.04% of its ionic conductivity, which is significantly worse than in advanced Li/Na electrolytes that reach values as low as 10^−4^‐10^−6^. The study also examined the extrinsic doping strategies (using aliovalent elements) to reduce harmful defects and tailor electronic properties.

Another group focused on kesterite‐based CZTSSe (Cu_2_ZnSn(S,Se)_4_) solar cells, which are promising alternatives to CdTe and CIGSe because they are made from earth‐abundant and non‐toxic elements. However, CZTSSe's highest efficiency came out to be 12.6%, which is much lower than CdTe (22.1%) and CIGSe (22.6%). The main issue was cation disorder, particularly Cu‐Zn antisite defects, due to the similar sizes and charges of Cu^+^ and Zn^2+^ ions. These defects created band tailing, which reduced the open‐circuit voltage (V_oc_). Even in the best CZTSSe cells, the V_oc_ deficit (Eg/q—V_oc_) remained over 0.6 eV with limited performance. To reduce this order, it was suggested that there is a need to control the synthesis conditions of solar cells, like sulfurization temperature and cooling rates, along with the use of isoelectronic cation substitution (e.g., replacing Zn with larger Cd or Mn) to reduce defect formation and exploring alternative structures beyond the traditional zinc‐blende framework. It was reported that the most successful CZTSSe films so far come from the hydrazine‐based solution process, giving micrometer‐sized grains and fewer secondary phases. Even so, full control over Cu‐Zn ordering remains difficult, especially since current thermal processing promotes random cation placement [112].

Doping is another important method used to modify the properties of ETM‐based chalcogenides. Doping involves intentionally introducing foreign atoms into the crystal lattice to alter its electronic, optical, or catalytic characteristics. By carefully selecting the type and concentration of dopants, we can fine‐tune the behavior of these materials for specific applications. A research explored how doping the edges (instead of surfaces) of 2D Bi_2_Te_3_ nanoplates with metals like Cu enhanced the thermoelectric (TE) performance of the system [113]. These 2D materials are already good TE candidates due to their low thermal conductivity and high carrier mobility. But performance often suffers when doping increases the carrier concentration (n) because it typically reduces the Seebeck coefficient (S) and mobility (µ). So, by targeting the reactive edges of the nanoplates, this study showed that it is possible to maintain high mobility even as carrier density increases. This “topological doping” created clean, atomically ordered interfaces that supported efficient charge exchange near the edge‐localized topological surface states of Bi_2_Te_3_. As a result, the power factor (PF = σS^2^) improved significantly, as the Cu‐doping boosted the PF by 8× compared to undoped Bi_2_Te_3_. The PF of doped nanoplates exceeded 5 mW/mK^2^ at room temperature, which is competitive with commercial thermoelectrics. Temperature‐dependent conductivity measurements suggested that local band bending at the doped edges acted as an energy filter, improving the overall selective charge injection and carrier scattering also did not increase much with doping. When elements such as rhenium are added to MoS_2_, they introduce additional charge carriers, enhancing the material's electrical conductivity [35, 39, 110, 114, 115, 116, 117]. This modification makes MoS_2_ more suitable for applications in transistors, sensors, and other electronic devices. Similarly, doping tungsten diselenide (WSe_2_) with certain elements can enhance its catalytic efficiency, making it more effective for use in chemical reactions such as hydrogen evolution reactions (HER) [42, 43, 110, 114, 117].

Strain engineering is another approach used to modify the properties of ETM‐based chalcogenides. By applying mechanical strain to the material, one can change the bond lengths and angles between atoms, which can alter the electronic band structure. This technique is particularly useful for tuning the band gap of semiconducting chalcogenides, allowing for better control over their electrical and optical properties [119, 120]. Strain can be introduced by growing thin films of these materials on substrates with different lattice constants or by applying external pressure. The ability to control material properties through strain engineering opens up new possibilities for the development of flexible electronics, stretchable sensors, and tunable optoelectronic devices. The interplay between phase transitions, defects, doping, and strain engineering provides a rich platform for designing ETM‐based chalcogenides with tailored properties [121, 122, 123, 124, 125, 126, 127, 128]. Scholars across the globe continue to explore new ways to manipulate these materials to enhance their performance in energy storage, catalysis, and electronic applications. The study of phase transitions and material modifications in ETM‐based chalcogenides remains an active area of research, with ongoing efforts to discover new phases, optimize doping strategies, and refine strain engineering techniques.

**FIGURE 15:**
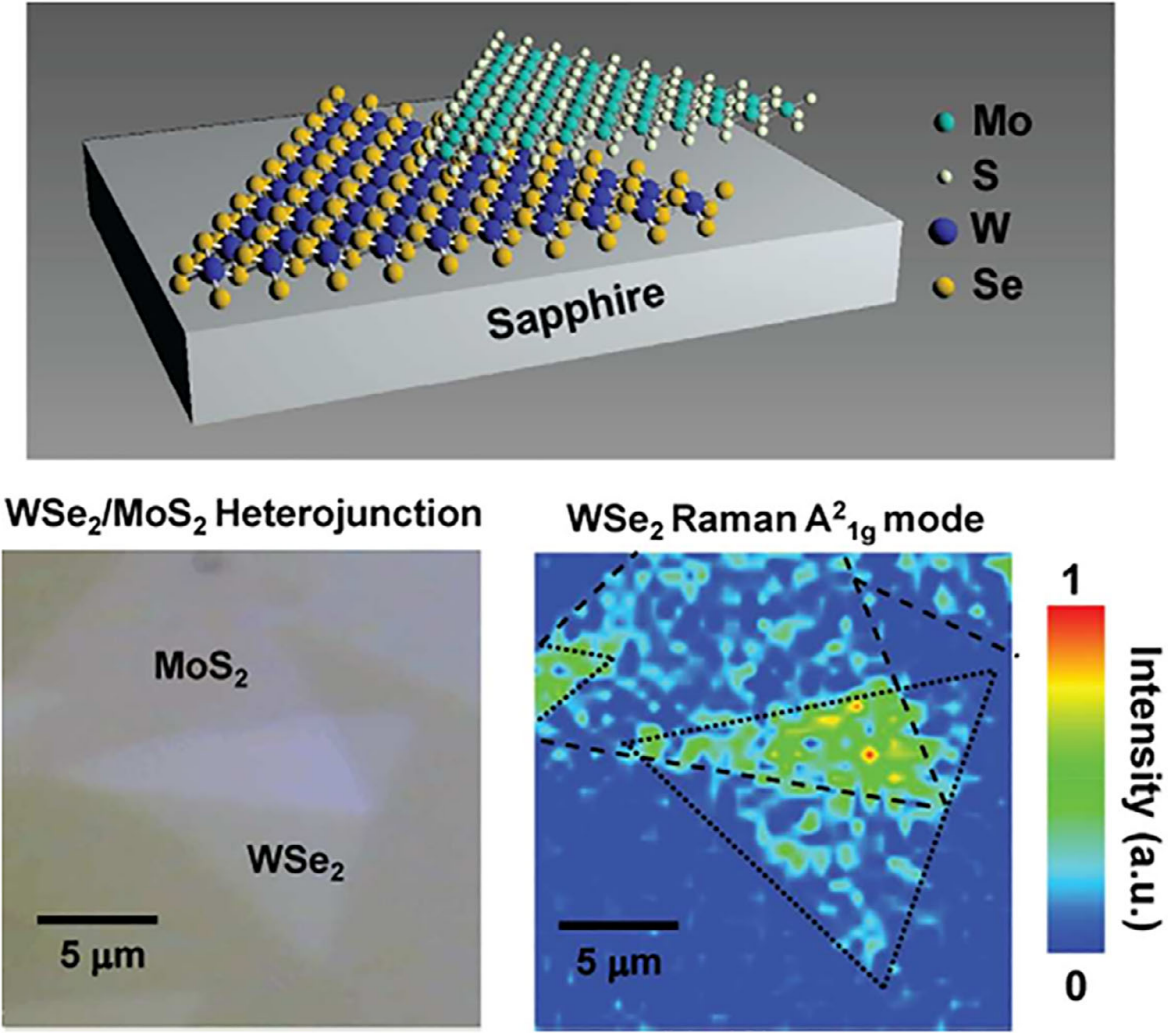
Representation and characterization of the vertical WSe_2_/MoS_2_ heterojunction. (on top) Schematic illustration of the vertical heterojunction formed by a monolayer of MoS_2_ deposited on top of a monolayer of WSe_2_ on a sapphire substrate, showing the arrangement of Mo, W, S, and Se atoms, (on bottom left) Optical microscopy image highlighting the stacked WSe_2_ and MoS_2_ regions, confirming the formation of the heterojunction and (on bottom right) Raman intensity map of the WSe_2_ A^2^_1_g mode, showing spatial variation in the vibrational response across the heterostructure. The enhanced/diminished Raman intensity indicates differences in layer thickness, stacking quality, and interlayer coupling within the heterojunction. Reproduced with permission [118]. Copyright 2014, ACS

Recent research on chalcogenides has also focused on heterostructures, where different layers of chalcogenide materials are stacked together to create new materials with improved properties. These heterostructures allow for combining different crystal structures, leading to materials with superior electronic, optical, and mechanical characteristics [129, 130, 131, 132, 133, 134]. The main advantage of heterostructures is that they allow for precise control of material properties at the atomic level. Some studies have reported that molybdenum disulfide (MoS_2_) and tungsten diselenide (WSe_2_) can be stacked together to create a heterostructure with unique electronic properties that differ from those of the individual materials. The interaction between monolayers of MoS_2_ and WSe_2_ was reported, and how they form van der Waals heterostructures when stacked vertically was studied (as shown in Figure 15) [118]. Initially, the layers were weakly coupled, but after thermal annealing at 300°C for 4 h in a H_2_/Ar environment, a strong interlayer coupling developed between the two materials. Using Raman spectroscopy and photoluminescence, it was shown that there is an enhancement in the interaction between MoS_2_ and WSe_2_. The Raman peak A^2^_1g_ for WSe_2_ at 309 cm^−1^, which is inactive in single layers, appeared after annealing, indicating interlayer hybridization. Similarly, for MoS_2_, E′ and A′_1_ modes shifted (red/blue), and a new E″ mode appeared around 284 cm^−1^. These changes confirmed strain‐induced coupling effects. The PL spectra also showed new exciton peaks from interlayer electron‐hole recombination, which weren't visible before annealing. Simulations using DFT also verified these experimental results, along with the AFM images showing that the heterostructures remained structurally clean and intact after annealing, with thicknesses of ∼0.6–0.7 nm per monolayer. This coupling allowed the formation of type‐II band alignment, which can be ideal for photovoltaics and photodetectors.

Ambient‐pressure chemical vapor deposition (APCVD) was used to report a simple and scalable method to grow few‐layered tungsten diselenide (WSe_2_) nanosheets directly on tungsten (W) foil [135]. The process used elemental selenium and argon gas without toxic byproducts, avoiding complex precursors like H_2_Se. Optimized reaction conditions at 900°C for 1 h with 200 sccm argon flow and 1 g selenium produced uniform, high‐quality 2H‐WSe_2_ films. Characterizations confirmed a layered crystal structure with high purity and nanosheet thickness between 50–80 nm. The synthesized WSe_2_ displayed a bandgap of 1.6 eV and an interlayer spacing of 0.648 nm, which is ideal for lithium‐ion intercalation. In lithium‐ion battery tests, exfoliated WSe_2_ showed a high and stable discharge capacity of 550 mAh/g at 250 mA/g after 50 cycles, maintaining its structure over 500 cycles. This is higher than the typical graphite anodes (∼372 mAh/g). The results indicated excellent long‐term stability and reversibility, as well as the layered structure helped in preventing the volume changes during cycling, which degrades the battery materials. The approach was scalable to 4 cm × 4 cm foils, showing potential for industrial use. Investigation on how the interaction between stacked 2D layers (MoS_2_, WS_2_, and WSe_2_) changes with temperature, and its effect on their electronic properties has also been explored. Heterostructures like MoS_2_‐WS_2_, MoS_2_‐WSe_2,_ and WS_2_‐WSe_2_ were synthesized using a film transfer method, and then analyzed them using photoluminescence, Raman spectroscopy, and density functional theory. It was observed that by increasing the interlayer spacing, the heterostructures can be turned from indirect to direct‐bandgap semiconductors (like MoS_2_‐WS_2_) by improving their optical activity. The MoS_2_‐WS_2_ system showed the strongest tunability as its bandgap shifted from indirect (VBM at Γ, CBM at K) to direct (both at K) with slight changes in spacing (∼5.155 to 7.155 Å). This transformation was much weaker in MoS_2_‐WSe_2_ and WS_2_‐WSe_2_, where valence band maxima at Γ were less sensitive to spacing. The heterostructures formed type‐II band alignments, enabling spatial separation of electrons and holes, which are useful for photodetectors and solar cells [136].

**FIGURE 16:**
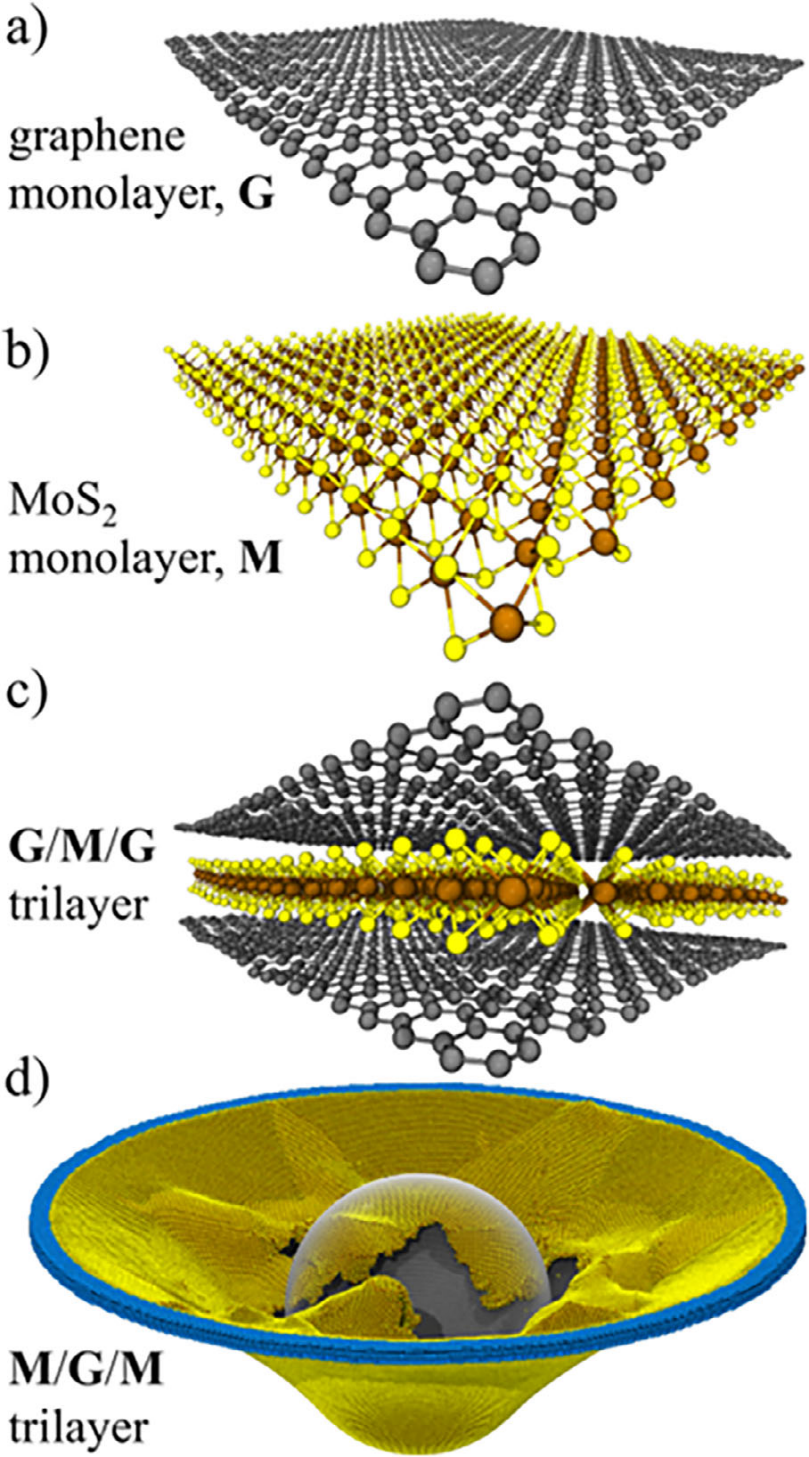
Atomistic schematics of (a) graphene (G) and (b) MoS_2_ (M) mono layers, (c) a graphene‐MoS_2_‐graphene (G/M/G) trilayer heterostructure. Carbon, molybdenum, and sulfur atoms are colored gray, brown, and yellow, respectively and (d) the M/G/M trilayer during nanoindentation. The clamped boundary is colored blue, and the nanoindenter is shown as a transparent sphere. Reproduced with permission [137]. Copyright 2015, AIP.

An investigation on how stacking graphene and MoS_2_ affects the mechanical strength by using molecular dynamics nanoindentation has been reported (see Figure 16) [137]. Pure graphene had a Young's modulus of 1.05 TPa, while MoS_2_ was much lower at 0.16 TPa. When combined in bilayers (G/M or M/G), the modulus increased to ∼0.5 TPa, showing that graphene strengthens the structure regardless of stacking order. The bending modulus of these bilayers was over 100 eV, which is much higher than MoS_2_ alone (8.5 eV), and the ultimate strength reached ∼120 GPa for bilayers, which was only 36 GPa for MoS_2_. Trilayers like G/M/G had even greater stiffness (modulus 0.68 TPa, bending modulus ∼727 eV) and better mechanical resilience, as well as the heterostructures could stretch up to 15% before breaking, which is more than that of MoS_2_ alone (∼10%). So it can be said that by stacking materials with different properties, one can create structures that exhibit unusual quantum behaviors, such as topological insulator states or superconductivity.

## 4 Applications of ETM‐Based Chalcogenides

**FIGURE 17:**
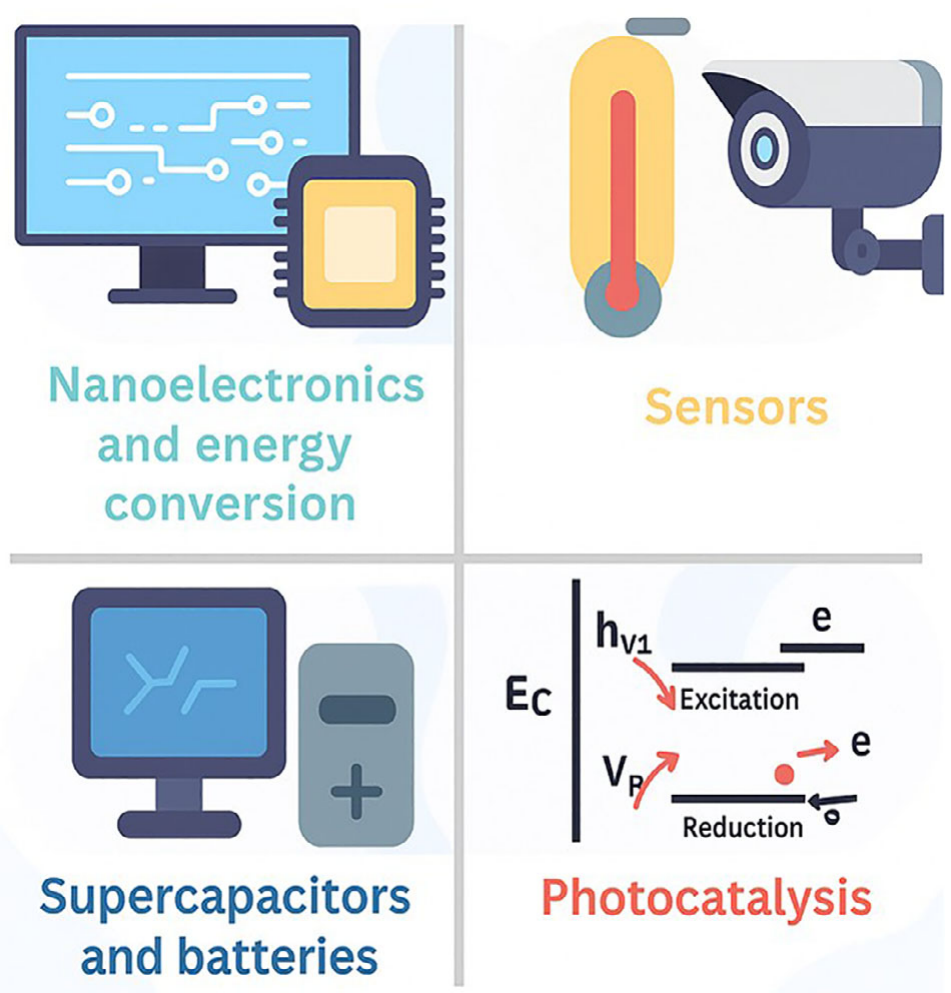
Schematic illustrations highlight the diverse technological fields where ETM‐based chalcogenides are widely utilized. These include nanoelectronics and energy conversion, where their tunable electronic structures enable high‐performance transistors, logic devices, and thermoelectric systems; sensors, leveraging their surface reactivity and high sensitivity for chemical, environmental, and optical detection; supercapacitors and batteries, where their high conductivity and redox activity support improved charge storage and cycling stability; photocatalysis, where band‐structure shows excitation and reduction processes that drive light‐induced catalytic reactions.

ETM‐based chalcogenides find applications in various fields, including nanoelectronics, energy conversion, as well as sensing and storage technologies like batteries and supercapacitors, as shown in Figure 17 [138, 139, 140, 141]. Homogeneous MoS_2_ field‐effect transistors (FETs) offer effective performance due to their remarkable reproducibility and uniformity across 2‐inch wafers [142, 143]. A statistical analysis of 30 4L MoS_2_ devices revealed that 60% of the fabricated devices exhibited mobilities ranging from 5 to 10 cm^2^V^−1^s^−1^, with an average mobility of 6.2 cm^2^V^−1^s^−1^ [144]. To achieve sensitive and stable gas sensing properties for NO_2_ gas, MoSe_2_ films were intentionally doped with Nb [145, 146]. The enhancement in gas response and stability can be ascribed to the cooperative influence of MoSe_2_ and Nb dopants, which effectively suppressed grain growth in MoSe_2_, leading to an augmentation in both the number of grains and the surface‐to‐volume ratio [12]. In the realm of energy conversion, ETM‐based chalcogenides serve as catalysts, effectively lowering the overpotential in electrochemical reactions [147]. Notably, the active sites situated at the edges of these chalcogenides play a pivotal role in catalysis [148, 149].

Furthermore, ETM‐based chalcogenides possess unique attributes that make them well‐suited for use as electrodes in batteries and supercapacitors [150, 151]. This suitableness results from the 2D sandwiched layers' strong covalent bonds, which enable them to store energy efficiently [152]. Moreover, ETM‐based chalcogenides have weak van der Waals connections between these interlayers, resulting in layered structures that are stacked loosely. Because of this, metal ions can easily intercalate between the layers of these chalcogenides without significantly altering their volume or causing structural deformations [153]. Certain ETM‐based chalcogenides are characterized as semiconductor materials featuring wide band gaps. These materials can generate photo‐excited electron‐hole pairs, leading to the formation of highly oxidative radicals [154, 155]. These radicals, in turn, facilitate the photocatalytic degradation of sizable molecules, including organic dyes [156, 157]. One of the most straightforward methods to assess the electrical properties of a material is to incorporate it into an electronic device, such as a field‐effect transistor (FET) [158, 159]. In this multi‐terminal device, the current is injected by applying a voltage between the source and drain electrodes, while the charge density is modulated and the Fermi level adjusted using the gate electrode.

### 4.1 Electronic Applications

In the last decade, Radisavljevic et al. [160] introduced the first field‐effect transistor (FET) based on a 2D ETM‐based chalcogenide material, employing a HfO_2_ top‐gate dielectric. The device showcased a room temperature ON/OFF ratio exceeding 10^8^, with OFF‐state currents smaller than 25 fA/µm. Typically, an ON/OFF ratio of 10^6^ and an OFF‐state current of around 1 nA/µm are deemed acceptable for digital electronic circuits. This groundbreaking study highlighted the potential of 2D materials, beyond graphene, to achieve electronic devices with remarkable performance characteristics. Studies have demonstrated that the semiconducting nature, atomically thin body, and absence of dangling bonds in ETM‐based chalcogenides enable excellent electrostatic gate control and reduced short‐channel effects, making them highly attractive for scaled nanoelectronic devices. In particular, MoS_2_, WS_2_, and related transition metal dichalcogenides (TMDs) exhibit thickness‐dependent band gaps and relatively high carrier mobilities, which allow systematic tuning of device performance through layer engineering and dielectric integration [161, 162]. These characteristics have enabled the realization of logic inverters, flexible electronics, and low‐power electronics operating at room temperature.

In addition to their compelling electronic applications, the ETM chalcogenide semiconductors exhibit favorable mechanical properties [163, 164]. Initial measurements of the stiffness and breaking strength of single‐layer (SL) and bilayer (BL) MoS_2_ were conducted by suspending MoS_2_ membranes on perforated substrates and deforming them using an AFM tip. These experiments revealed that MoS_2_ has a Young's modulus of approximately 270 GPa, surpassing that of steel (about 205 GPa). The breaking strength of SL and BL MoS_2_ ranged between 6% and 11% of their respective Young's modulus, which approaches the upper theoretical limit of a material's breaking strength, highlighting the intrinsic properties of its interatomic bonds [165]. The combination of high mechanical robustness and electronic tunability has also enabled the development of strain‐engineered electronic devices, where controlled mechanical deformation is used to modulate band structure, carrier mobility, and excitonic behavior. Such strain‐tunable electronics are particularly relevant for flexible and wearable device architectures based on ETM chalcogenides [166].

**FIGURE 18:**
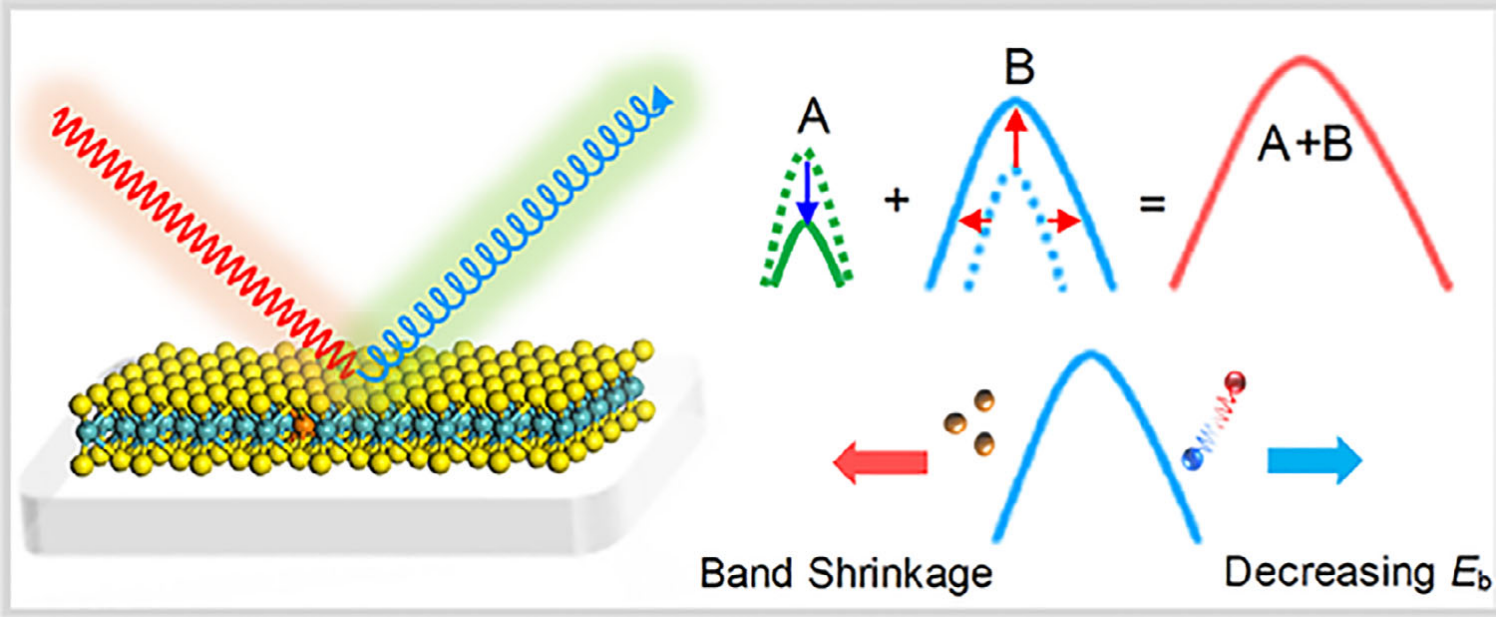
Schematic illustration of the n‐type to p‐type transition in the synthesized semiconductor films, showing how external influences alter the electronic band structure: mechanisms A and B combine to cause band shrinkage and reduce the electron binding energy (E_b_). These changes shift the dominant charge carriers from electrons to holes, resulting in a transformation from n‐type to p‐type semiconductor behavior. Reproduced with permission [167]. Copyright 2021, ACS.

In 2017, Gobadi [168] used molecular dynamics simulations to compare the mechanical behavior of multilayer MoS_2_ and graphene/MoS_2_ heterostructures under uniaxial and compressive strain. The graphene/MoS_2_ structure had much higher stiffness, with a Young's modulus of 446.7 GPa, compared to 152.8 GPa for MoS_2_ alone, due to graphene's inherent strength. Under normal compressive strain, the vertical stiffness (C_33_) of the heterostructure reached 199.5 GPa, far greater than 51.8 GPa for MoS_2_. Additionally, stacking order mattered, i.e., high‐energy AA_3_/AB_3_ configurations reduced mechanical strength. The graphene layers also helped the structure resist thermal vibrations. A study explored how doping MoS_2_ with niobium (Nb) changes its optical and electronic behavior, transforming it from an n‐type to a p‐type semiconductor [167]. Using a two‐step method, wafer‐scale Mo_1‐x_Nb_x_S_2_ films with Nb concentrations up to 7.6% were produced. As Nb increased, the material's photoluminescence showed a redshift in exciton peak A and a blueshift in peak B, indicating impurity‐induced band shrinkage and reduced exciton binding energy. The peaks eventually merged into a broad A+B peak at 1.89 eV, as evident from Figure 18. Ellipsometry confirmed this spectral behavior and showed dielectric function changes tied to doping. Simulations revealed that new valence bands from Nb caused multiple transitions, broadening peak B. A field‐effect transistor built using this material confirmed p‐type conduction with a hole mobility of 0.813 cm^2^/Vs and contact resistance of ∼10^4^ Ωµm. Beyond pristine TMDs, intentional doping and alloying have emerged as powerful strategies to tailor the electronic behavior of ETM‐based chalcogenides. For instance, substitutional doping introduces impurity states near the band edges, enabling controlled n‐type or p‐type conduction and improved contact engineering in FETs. Such approaches are essential for realizing complementary metal oxide semiconductor (CMOS) architectures using 2D materials [169].

Kuc & Heine [170] explored how alloying MoS_2_ monolayers with W, Nb, or Se affected their stability and electronic properties using density functional theory. W and Se formed stable solid solutions with MoS_2_, while Nb got clustered and caused phase separation. Notably, W‐doping (e.g., Mo_0.05_W_0.5_S_2_) resulted in a bandgap between 1.84 eV (MoS_2_) and 2.01 eV (WS_2_), and a valence band spin‐orbit splitting of 279 meV, which is intermediate between MoS_2_(150 meV) and WS_2_ (425 meV). Alloying by Se allowed a bandgap tuning between 1.53 and 1.84 eV and introduced spin‐splitting of ∼170–180 meV. It was also observed that Nb‐doping caused metallic behavior even at 6.25% due to the formation of localized states. Furthermore, MoW and MoSe alloys maintained a direct bandgap, suitable for optoelectronics and spintronics. So, it was concluded that MoS_2_‐based alloys enable fine‐tuning of electronic and spintronic properties, making them promising for advanced 2D material applications. A recent study predicted a new class of 2D metal‐rich chalcogenide monolayers denoted by M_2_X (Hf_2_Se, Zr_2_Te, etc.) with intrinsic antiferromagnetic order and high Néel temperatures using first‐principles DFT calculations [171]. These structures, with an “anti‐structure” of MoS_2_, consisted of a chalcogen layer sandwiched between two metal layers, due to which they exhibited stable antiferromagnetic coupling between metal layers and ferromagnetic order within layers, mediated by super‐exchange interactions. Hf_2_Se showed a Néel temperature of 436 K, a magnetocrystalline anisotropy energy of 1630 µeV/cell, and a bandgap of 451.7 meV (HSE06). MAEs increased with heavier metal atoms, reaching up to 2228 µeV/cell for Hf_2_Te, and all the systems except Ti_2_Te at room temperature were thermodynamically stable. It was concluded that the combination of high Néel temperatures, large magnetocrystalline anisotropy energy, and semiconducting behavior positions M_2_X monolayers as excellent candidates for room‐temperature spintronic devices.

In parallel, increasing attention has been directed toward sesqui‐ and polychalcogenides, which exhibit electronic properties distinct from conventional dichalcogenides. Sesquichalcogenides such as Mo_2_S_3_ and V_2_S_3_ possess metal‐rich compositions, leading to higher intrinsic electrical conductivity and narrower band gaps compared to MoS_2_. These features reduce contact resistance and enhance charge transport, addressing key bottlenecks in TMD‐based electronics. Polychalcogenides, including Se‐ and Te‐rich compounds such as MoSe_2‐x_ and ReSe_2_, exhibit strong in‐plane anisotropy, reduced symmetry, and complex band structures that give rise to direction‐dependent carrier mobility and polarization‐sensitive transport. Such anisotropic electronic behavior is highly desirable for next‐generation logic devices, photodetectors, and neuromorphic electronics [172, 173, 174].

DFT and AIMD simulations on a novel 2D Hf_2_Cse_2_ monolayer were studied, combining features of MXenes and transition metal dichalcogenides, and the material's structural, mechanical, and electronic properties were evaluated. The optimized structure had a lattice constant of 3.45 Å, Hf‐Se and Hf‐C bond lengths of 2.67 and 2.37 Å, respectively, and a buckling height of 6.11 Å. Phonon dispersion and AIMD confirmed its dynamical and thermodynamic stability at 300 K while demonstrating isotropic elastic properties with Young's modulus of 167.28 N/m, shear modulus of 66.95 N/m, and Poisson's ratio of 0.249. Electronic band structure revealed metallic behavior, with Se p‐orbitals dominating near the Fermi level and C 2p states near the valence band. These findings suggest Hf_2_CSe_2_ is stable, conductive, and structurally robust, making it a promising candidate for applications in nanoelectronics, sensing, and possibly thermoelectrics [175]. Hf_2_SeC has also been studied using DFT, whose structure is found to be both dynamically and mechanically stable, with lattice constants a = 3.438 Å and c = 12.501 Å [176]. It exhibited a metallic nature with mixed ionic‐covalent bonding, which was confirmed through band structure and charge density mapping. It was discovered that Hf_2_SeC achieved a Chen hardness of 18.47 GPa and Young's modulus of 273 GPa, as well as being mechanically anisotropic and brittle, with a Pugh ratio of 0.72 and Poisson's ratio of 0.21. The thermal properties are promising for high‐temperature applications, including a Debye temperature of 640 K, a melting point of 1674 K, and a minimum thermal conductivity of 0.86 W/mK. It was concluded that its thermal expansion coefficient of 2.66 × 10^−5^K^−1^ at 300 K and stability under pressure and temperature make it a potential candidate for thermal barrier coatings and other extreme‐environment applications. These emerging sesqui and polychalcogenide systems expand the electronic materials landscape beyond conventional TMDs, offering new opportunities for high‐performance electronics, spintronics, and multifunctional devices operating under extreme conditions.

Table 1 shows the diversity of electronic properties across various ETM‐based chalcogenides, highlighting how composition and crystal phase influence key properties such as bandgap and carrier mobility. The electronic properties of ETM‐based chalcogenides are strongly governed by their stoichiometry, crystal symmetry, and dimensionality, which together determine orbital overlap, carrier transport pathways, and defect tolerance. ETM‐based chalcogenides, particularly MoS_2_, WS_2_, and WSe_2_, exhibit highly ordered layered structures composed of X‐M‐X trilayers held together by van der Waals forces. This structural motif results in strong in‐plane covalent bonding and weak interlayer interactions, enabling excellent electrostatic gate control and thickness‐dependent band structure modulation. As a consequence, monolayer ETM‐based chalcogenides display direct band gaps in the range of ∼1.5–2.1 eV, making them highly suitable for logic electronics, photodetectors, and optoelectronic devices [177, 178, 179]. In addition to favorable band gaps, these benefit from relatively low defect densities and symmetric crystal lattices, which support moderate‐to‐high carrier mobilities and high ON/OFF current ratios. These attributes explain why MoS_2_‐based field‐effect transistors have become the benchmark for 2D electronics. However, even within ETM‐based chalcogenides, significant variability exists: materials such as WSe_2_ enable ambipolar transport, while Re‐based dichalcogenides exhibit distorted 1T′ phases that introduce strong in‐plane anisotropy [180]. This anisotropy leads to direction‐dependent carrier mobility and optical response, opening opportunities for polarization‐sensitive and anisotropic electronic devices that are inaccessible with hexagonal 2H ETM‐based chalcogenides.

**TABLE 1:** Comparative summary of electronic properties of early transition‐metal chalcogenides, including ETM‐based chalcogenides, sesquichalcogenides, and polychalcogenides, highlighting crystal structure, bandgap, carrier characteristics, and representative electronic applications.

Class	Material	Phase	Bandgap (eV)	Carrier type / Mobility	Electronic properties	Applications	Refs.
TMD	MoS_2_	2H (monolayer)	1.8 (direct)	n‐type, 10–60 cm^2^/Vs	High ON/OFF ratio, tunable bandgap	FETs, sensors, photodetectors	[177]
TMD	WS_2_	2H	2.0	n‐type	Strong excitonic effects	Optoelectronics	[179]
TMD	WSe_2_	2H/3R	1.6	Ambipolar	Strong spin–orbit coupling	Logic devices, photonics	[178]
TMD	MoTe_2_	2H/1T′	1.1	Ambipolar	Phase‐change electronic behavior	Memory, switches	[181]
TMD	ReS_2_	Distorted 1T	1.4	n‐type, anisotropic	In‐plane anisotropic transport	Polarized photodetectors	[180]
Sesquichalcogenide	Fe_2_S_3_	Orthorhombic	0.9–1.1	p‐type	Defect‐rich electronic states	Sensors, catalysis	[182]
Sesquichalcogenide	Ti_2_S_3_	Layered	0.2–0.4	Low mobility	Narrow‐gap semiconductor	Memory devices	[183]
Sesquichalcogenide	Nb_2_Se_3_	Quasi‐1D	Semi‐metallic	Highly anisotropic	Charge density wave behavior	Nanoelectronics	[184]
Polychalcogenide	MoS_3_	Amorphous	Pseudo‐gap	Localized transport	High density of mid‐gap states	Neuromorphic electronics	[185]
Polychalcogenide	WS_3_	Amorphous	Disordered	Low mobility	Charge trapping, resistive switching	Memory, catalysis	[186]
Polychalcogenide	NbSe_3_	Chain‐like	Metallic	High conductivity	Charge density waves	Low‐dimensional electronics	[187]

Sesquichalcogenides represent a fundamentally different electronic regime. Their reduced chalcogen content leads to incomplete coordination of metal atoms, giving rise to chain‐like or ribbon‐type crystal structures rather than continuous 2D sheets. This lower symmetry produces narrow band gaps or semimetallic behavior, accompanied by a high density of intrinsic defects and localized electronic states. As a result, sesquichalcogenides typically exhibit lower carrier mobility and weaker gate modulation compared to conventional TMDs, limiting their applicability in conventional digital electronics [182, 183, 184]. However, these same characteristics enhance electronic coupling, charge localization, and defect‐mediated transport, making sesquichalcogenides attractive for applications such as resistive switching, sensing, and electrochemical devices where strong electronic interactions are desirable.

Polychalcogenides further extend this trend toward defect‐dominated electronic behavior. Their chalcogen‐rich compositions often result in amorphous or poorly crystalline structures containing chalcogen chains, vacancies, and disordered bonding networks. These features introduce mid‐gap states that reduce carrier mobility but significantly increase charge trapping and redox activity. Consequently, polychalcogenides generally exhibit indirect band gaps and limited suitability for high‐speed electronics [185, 187]. Nonetheless, their tunable conductivity and defect‐rich electronic structure make them promising candidates for emerging technologies such as neuromorphic computing, memory devices, and electrochemical transducers, where controlled charge trapping and gradual conductance modulation are essential.

From an application perspective, ETM‐based chalcogenides remain the materials of choice for high‐performance electronics and optoelectronics due to their clean band structures and scalable device integration. In contrast, sesqui and polychalcogenides occupy a complementary niche, offering electronically active defect states and unconventional transport mechanisms that are difficult to achieve in stoichiometric ETM‐based chalcogenides. The future of electronic materials in this family is therefore likely to lie in hybrid architectures, where ETM‐based chalcogenides provide efficient charge transport while sesqui or polychalcogenides introduce functional electronic states, enabling multifunctional devices that bridge logic, memory, and sensing within a single material platform.

### 4.2 Catalytic Applications

It has been well established that chalcogenides exhibit excellent catalytic activity due to their tunable electronic structures and abundant active sites. Their layered structures enable efficient charge transfer and facilitate surface reactions. These materials are widely explored for applications in hydrogen evolution, oxygen reduction, and CO_2_ conversion reactions [188, 189, 190, 191, 192, 193]. 2D chromium chalcogenide nanosheets (Cr_2_S_3_, Cr_2_Se_3_, Cr_2_Te_3_) were synthesized using APCVD, and their electrocatalytic and electrical properties were examined [194]. It was observed that all nanosheets had an identical structure (P‐31c symmetry and the same exposed facets), allowing direct comparison. For the hydrogen evolution reaction (HER), Cr_2_S_3_ showed the best performance with the lowest overpotential and highest current density. It was noted that the electrical conductivity improved from Cr_2_S_3_ to Cr_2_Te_3,_ which means that better conductivity didn't lead to better HER activity. FET tests confirmed this electrical trend, while DFT simulations revealed that intrinsic catalytic activity was the key driver for HER performance. The flakes were about 8 nm thick and were grown under optimized temperature conditions (Cr_2_S_3_ at 720°C, Cr_2_Te_3_ at 750°C). These findings showed that designing electrocatalysts should focus more on catalytic surface chemistry than just improving electrical conductivity, especially in 2D materials.

**FIGURE 19:**
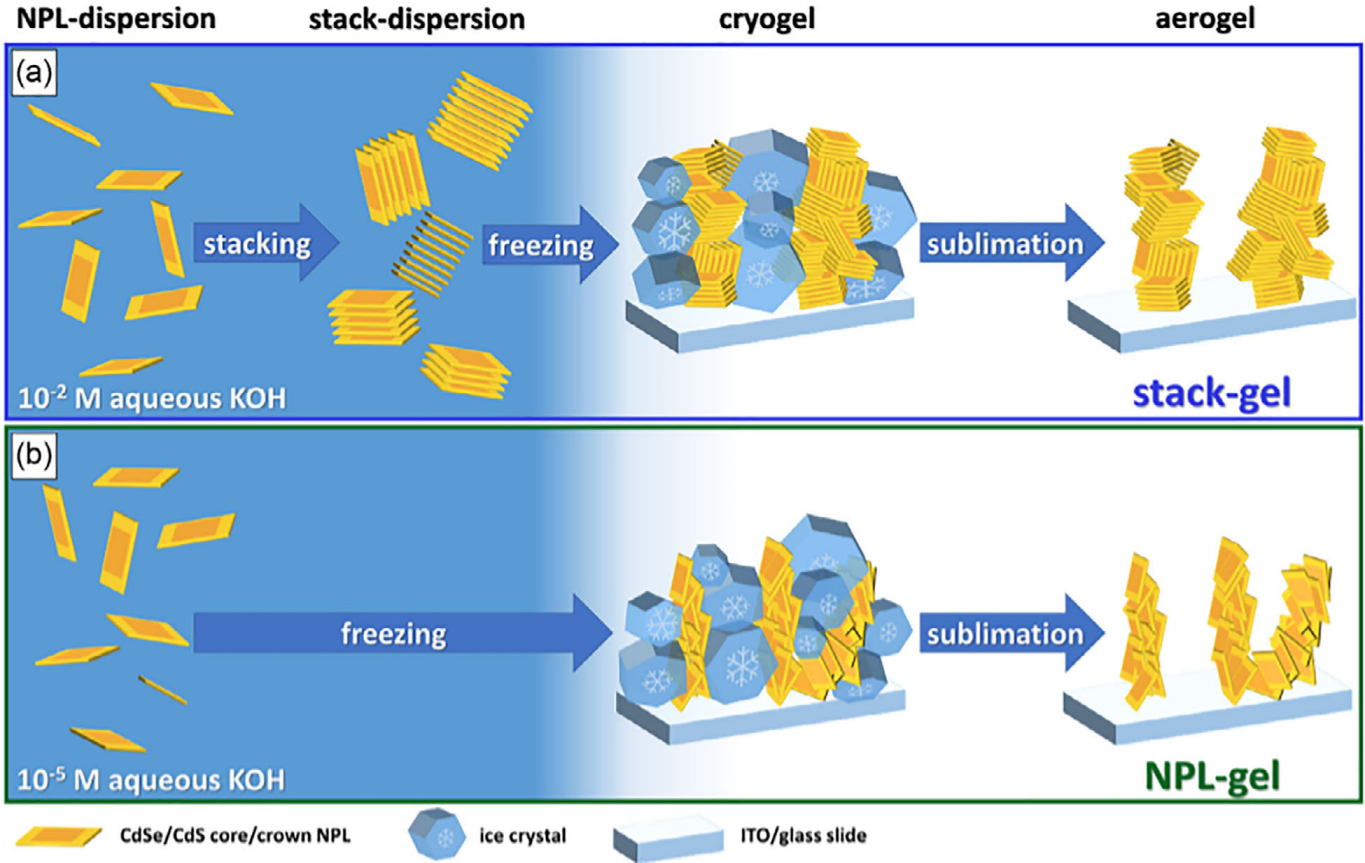
(a) Schematic description of the cryoaerogelation procedure of 11‐mercaptoundecanoic acid capped CdSe/CdS‐core/crown NPLs from aqueous KOH to form a stack‐gel and (b) an NPL‐gel; flash‐freezing of the particle solution with liquid nitrogen forms a cryogel, followed by sublimation of the ice crystals, generating so‐called cryoaerogels. Reproduced with permission [195] Copyright (2024) Wiley.

**FIGURE 20:**
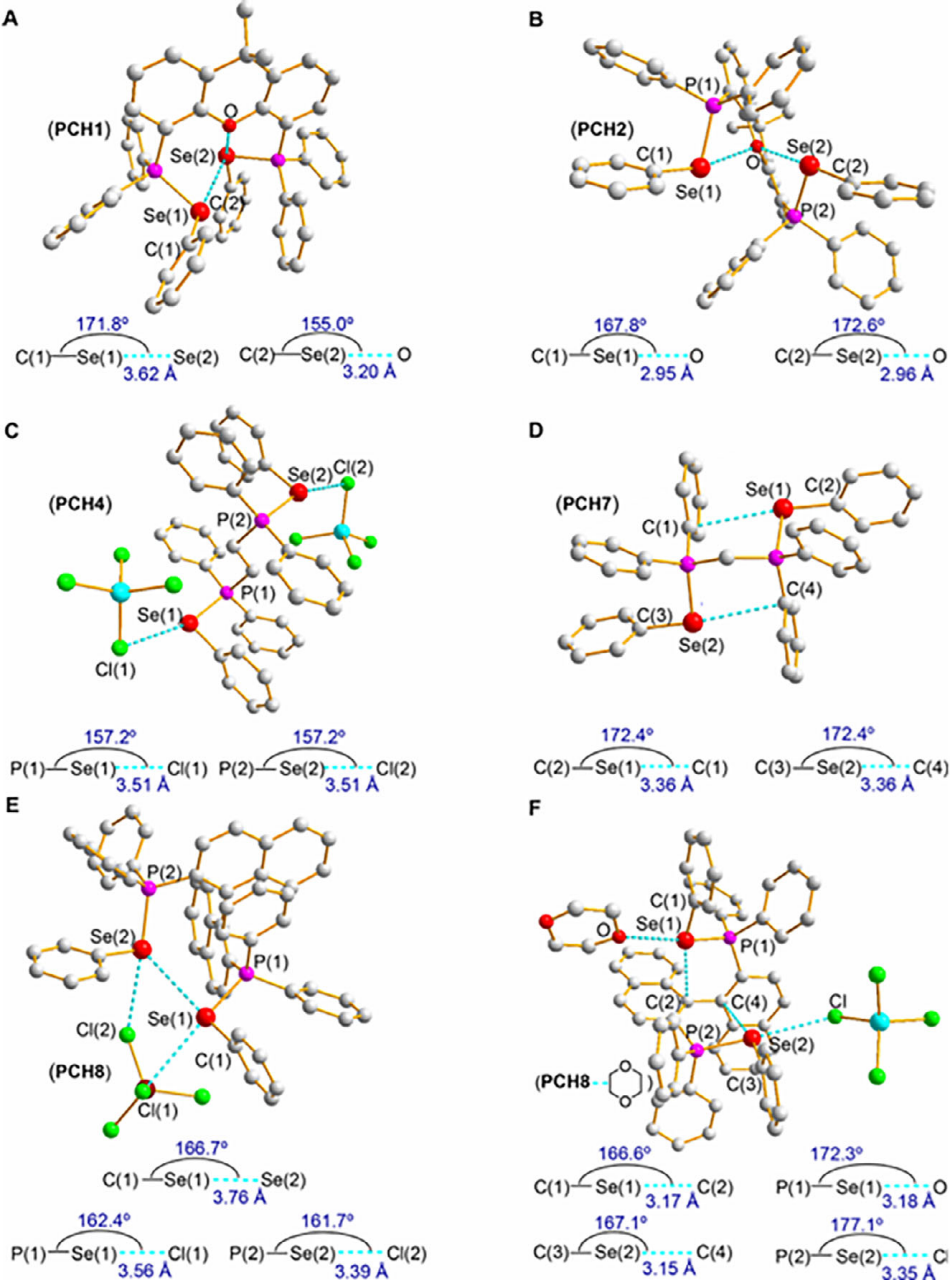
Chalcogen bonding properties of PCH catalysts (hydrogens, counteranions in PCH1,‐2, and ‐7, and one counteranion in PCH8 and PCH8‐dioxane were omitted for clarity in crystal structures). Reproduced with permission [188]. Copyright 2023, ACS.

Cadmium chalcogenide nanoplatelets (NPLs) were used for porous cryoaerogel networks, which preserved stacking while improving material accessibility for catalysis and sensing, as shown in Figure 19 [195]. Instead of traditional polymer encapsulation, they used cryoaerogelation, which involved stacking NPLs via antisolvent, flash‐freezing the dispersion with liquid nitrogen, and freeze‐drying to retain structure. The resulting aerogels maintained the NPL stacks and exhibited high porosity and surface area, verified by electron microscopy and gas physisorption. These features enable charge carrier transport through the stacks, confirmed via photoelectrochemical measurements. CdSe/CdS core/crown NPLs were used, with high PL quantum yield (up to 84%) and preserved exciton properties after gelation. The aerogels showed potential for photocatalysis, gas‐phase sensing, and other applications where material stability, surface access, and nanoscale properties are essential. Ajiboye et al. [196] highlighted the versatility of indium(III) complexes in organic catalysis, material science, and industry. Indium is less toxic and more water‐stable than metals like tin or zinc, making its complexes useful in aqueous and mild conditions. Indium(III) complexes are also excellent precursors for synthesizing chalcogenide nanoparticles like In_2_S_3_, In_2_Se_3_, and In_2_Te_3_, used in OLEDs, sensors, and solar cells. Zhao & Wang [188] introduced chalcogen bonding catalysis using phosphonium chalcogenides (PCH) as a novel strategy in organic synthesis (see Figure 20). Chalcogen bonding is a weak but directional interaction between chalcogen atoms (S, Se, Te) and electron donors. PCH catalysts exploited this to drive difficult reactions that traditional Lewis acid or base catalysts cannot easily handle. They enabled a ppm‐level catalyst loading for cyanosilylation of ketones and the efficient assembly of heterocycles from β‐ketoaldehydes and indoles. They also achieved Se‐π bonding to activate alkenes, resulting in selective cyclization and coupling reactions. The modular structure of the catalyst allowed fine‐tuning of catalytic activity through substitutions on either the chalcogen or phosphonium part. This approach solved the reactivity and selectivity challenges in reactions like the Rauhut‐Currier reaction, making it a promising platform for expanding noncovalent catalysis beyond hydrogen and halogen bonding. A recent research presented a series of indane‐based chiral aryl chalcogenide catalysts designed for asymmetric electrophilic reactions. These catalysts combined chiral indane backbones with sulfide or selenide groups, allowing for controlled reactivity and high enantioselectivity. By adjusting substituents on the aryl ring, their Lewis basicity and steric hindrance were tuned to match various substrates. These catalysts enabled reactions such as enantioselective CF_3_S‐lactonization, aminocyclization, and chlorocarbocyclization with excellent yields and stereoselectivity. The CF_3_S‐lactonization of olefinic acids gave access to seven‐membered rings with >90% enantiomeric excess (ee). The catalysts also worked for Friedel‐Crafts type electrophilic chlorination, constructing P‐chirogenic molecules. Their bifunctional binding mode is proposed to stabilize chiral intermediates and guide the reaction path [189]. CuInS_2_ and CuInSe_2_ nano/microparticles were synthesized without using any surfactants or polymer coatings, using a simple one‐step reaction with LiBH_4_ under controlled conditions. The method used in this study is rare because it doesn't need templates or extra steps to make complex metal compounds. It was also shown that these CuInS_2_ particles worked well as catalysts. They helped in joining substituted phenylacetylenes and dimerizing 1,3‐diynes efficiently at room temperature in acetonitrile, using DBU as a base. These reactions finished in 4–7 h with up to 97% yield, and it was noted that the catalyst remained effective over 5 cycles, which is attributed to its clean and accessible surface [197].

Another group synthesized new sulfur and selenium ligands and their complexes with group 10 metals (Pd, Pt). It was reported that the ligand 2‐(N‐(diphenylphosphino)amino)‐4‐methylpyridine was oxidized with sulfur or selenium to form chalcogenides, then reacted with PdCl_2_ and PtCl_2_ to form stable cis‐MCl_2_(P, N) complexes. Pd(II) complex (compound 4) was tested as a pre‐catalyst in the Suzuki cross‐coupling reaction and showed efficient performance. Crystallographic studies confirmed the square planar geometry of the Pt(II) and Pd(II) centers. The ^31P NMR showed key peaks at 23.66 ppm (Pd complex) and 48.06 ppm (Pt complex), while IR showed P‐S and P‐Se vibrations at 640 and 543 cm^−1^, respectively. The study highlighted the strong chelating ability of the P, N ligand and its ability to form stable five‐membered metallacycles. The Pd(II) complex was found to be a good pre‐catalyst for Suzuki reactions, suggesting potential applications in organometallic catalysis [198]. 2D Cu_2_MoS_4_ nanodots (CMS NDs) were developed with enzyme‐like peroxidase activity for treating bacterial wounds [199]. The nanodots (∼4 nm) were synthesized using a microwave‐assisted method and showed strong activity in acidic conditions (pH < 5.5). It was noted that when they were combined with H_2_O_2_, they generated hydroxyl radicals (‐OH) and achieved >2 log bacterial inactivation against both *E. coli* and MRSA. CMS NDs were renal‐clearable and showed good biocompatibility in mice. In vivo tests on MRSA‐infected wounds in mice showed significant healing with no harm to healthy tissue. Their enzyme‐like activity was spatially controlled, active only in infected areas with acidic microenvironments, which helped in reducing damage to the normal cells. Another group developed a copper‐molybdenum chalcogenide catalyst (CuMo_2_S_3_) to enhance the degradation of antibiotics in water using peroxymonosulfate (PMS). The catalyst demonstrated superior degradation of tetracycline, achieving a rate constant of 0.053 min^−1^, significantly outperforming CuS + PMS (0.022 min^−1^) and MoS_2_ + PMS (0.007 min^−1^). This was attributed to the presence of electron‐rich MoX and SX sites, which helped in regenerating active Cu^+^ centers continuously. The catalyst also showed broad pH tolerance (3‐11), stability in real water samples, and resilience to interfering substances like humic acid. With a BET surface area of 17.3 m^2^/g and good porosity, it offered efficient contact with pollutants. The proposed mechanism involved the generation of multiple reactive oxygen species (‐SO^4−^, ‐OH, ‐O^2−^, ^1^O_2_) supported by EPR and LC‐MS.

**FIGURE 21:**
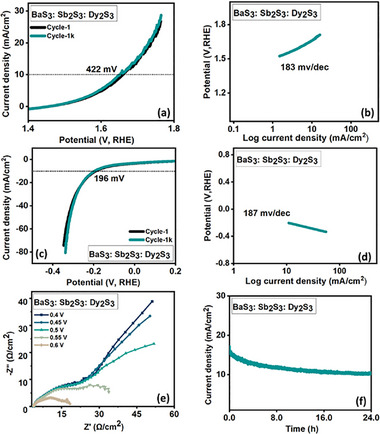
Electro‐catalytic characteristics of the semiconducting BaS_3_: Sb_2_S_3_: Dy_2_S_3_ STC hetero‐system, (a–d) LSV polarization curves with associated Tafel slopes, and (e,f) stability‐illustrative CA and Nyquist plots. Reproduced with permission [200]. Copyright 2025, Elsevier.

A ternary chalcogenide (BaS_3_: Sb_2_S_3_: Dy_2_S_3_) was synthesized using a single‐source precursor method for energy storage and electrocatalysis [200]. The synthesized chalcogenide had a bandgap of 3.73 eV and a crystallite size of 18.1 nm, with high crystallinity. It also showed excellent supercapacitor performance with a specific capacitance of 769.82 F/g and a power density of 11,608.85 W/kg, which indicates fast charge transfer and high stability. In electrocatalytic tests, it achieved an oxygen evolution reaction (OER) overpotential of 422 mV and a Tafel slope of 183 mV/dec, while hydrogen evolution reaction (HER) performance showed 196 mV overpotential and 187 mV/dec Tafel slope. This superior electrochemical performance (as shown in Figure 21) was attributed to its porous morphology and rich redox chemistry from multiple metal sites. It is a strong candidate for applications in hybrid energy storage devices and water splitting technologies.

The comparative data summarized in Table 2 highlight clear structure‐composition performance relationships governing the hydrogen evolution reaction (HER) and oxygen evolution reaction (OER) activity of early transition metal‐based chalcogenides and hybrid catalysts. For HER, catalysts incorporating transition metal nitrides, phosphides, and sulfides supported on conductive substrates, such as Ni foam, carbon frameworks, or Mxenes, consistently exhibit low overpotentials, indicating fast reaction kinetics and efficient charge transport. In particular, Ni‐ and Mo‐based systems such as NiMoN@Ni foam and Pt/ReS_2_ demonstrate overpotentials as low as 20–40 mV at 10 mA cm^−2^, which can be attributed to optimized hydrogen adsorption free energy (ΔG_H_∼0) and the presence of abundant exposed active sites. These materials benefit from strong metal‐chalcogen coupling and metallic or semi‐metallic conductivity, which collectively reduce electron‐transfer resistance during HER. Whereas, MoS_2_‐based catalysts dominate the table due to their well‐understood edge‐site‐driven catalytic mechanism, where sulfur edge sites act as the primary active centers for proton adsorption and hydrogen evolution.

**TABLE 2:** Comparison of electrocatalytic performance of chalcogenide‐based catalysts for hydrogen evolution (HER) and oxygen evolution reactions (OER) under different electrolytes, summarizing overpotential values at benchmark current densities and associated material systems.

Catalyst(s)	Target reaction	Electrolyte	Overpotential (mV at 10 mA/cm^2^)	Refs.
NiVN@Ni foam	HER	1 M KOH + 0.6 M NaCl	37	[97]
NiCrN@Ni foam	HER	1 M KOH + 0.6 M NaCl	115	[97]
20% Pt/C@Ni foam	HER	1 M KOH + 0.6 M NaCl	75	[97]
NiMoN@Ni foam	HER	1 M KOH + 0.5 M NaCl	20	[206]
Ni‐NiO‐Cr_2_O_3_@Ni foam	HER	1 M KOH + 0.5 M NaCl	40	[207]
Cu_2_S@Ni@CF	HER	1 M NaOH + 0.5 M NaCl	50	[208]
Ni_2_P‐Fe_2_P@Ni foam	HER	1 M KOH + seawater	220	[209]
Ni‐SA/NC	HER	1 M KOH + seawater	139	[210]
U‐CNT‐900	HER	buffered seawater (pH 7.0)	250	[211]
MoP700	HER	seawater (pH 7.8)	142	[212]
Ni_5_P_4_@Ni_2+δ_O_δ_ (OH)_2δ_@Ni foam	HER	seawater (pH 7.0)	144	[213]
Mn‐NiO‐Ni@Ni foam	HER	seawater (pH 8.2)	170	[214]
NiCoN/Ni_x_P/NiCoN	HER	seawater (pH 7.2)	165	[215]
Se‐MoS_2_	HER	0.5 m H_2_SO_4_	132	[216]
Co/Se‐MoS_2_	HER	0.5 m H_2_SO_4_	104	[216]
CoS_2_/MoS_2_/Gr	HER	1 m KOH	53	[217]
ReS_2_/ReO_2_/Gr/polyimide	HER	0.5 m H_2_SO_4_	150	[218]
Ru/2H‐MoS_2_	HER	0.5 m H_2_SO_4_	168	[219]
Ultrathin 1T/2H MoSe_2_	HER	0.5 m H_2_SO_4_	85	[220]
Ni‐doped ReSe_2_	HER	1 m KOH	109	[221]
Cu_7_S_4_/CuS_2_/NiS_2_	HER	0.5 m H_2_SO_4_	269	[222]
MXene/MoS_2_	HER	0.5 m H_2_SO_4_	69	[223]
CuS/CoS_2_	HER	1 m KOH	85	[224]
Cu_2_O/Cu_2_Se	HER	1 m KOH	52.9	[225]
Pt/ReS_2_	HER	0.1 m HClO_4_	20	[226]
2D PdTe_2_	HER	0.5 m H_2_SO_4_	76	[227]
NiFe‐60/Co_3_O_4_@Ni foam	OER	1 m KOH	190	[228]
Vacancy CoTe_2_	OER	1 m KOH	241	[229]
CoS_2_/MoS_2_/Gr	OER	1 m KOH	255	[217]
(NiFeCoMn)_3_S_4_	OER	1 m KOH	289	[230]
Co,Nb‐MoS_2_/TiO_2_	OER	1 m KOH	260	[231]
Co‐Ru‐MoS_2_	OER	1 m KOH	308	[232]
CrO_x_/CuS	OER	1 m KOH	190	[233]
N‐doped Ni_3_S_2_/CoS_2_	OER	1 m KOH	245	[234]
Fe‐doped CoSe_2_	OER	1 m KOH	220	[235]
Rh‐Co_3_S_4_/CoOx	OER	1 m KOH	242.8	[236]
CoTe_2_/CoP	OER	1 m KOH	260	[237]
Fe‐NiO/NiS_2_	OER	1 m KOH	270	[238]
N,P‐doped Co_9_O_8_/CoS_2_/Co_1‐x_S	OER	1 m KOH	285	[239]
CuS/Cu_2_S	OER	1 m KOH	192	[240]
Zn‐doped CoS_2_	OER	1 m KOH	248	[241]
Cu_2_S/Ni_3_S_2_	OER	1 m KOH	237 at 100 mA/cm^2^	[242]
NiCo LDH/NiCoS	OER	1 m KOH	207	[243]
CoOOH/Co_9_S_8_	OER	1 m KOH	240	[244]
CoTe_2_/NiTe_2_	OER	1 m KOH	280	[245]
RuSe_2_/CoSe_2_	OER	1 m KOH	200	[246]
CoSe_2_/NiSe_2_	OER	1 m KOH	160	[247]

Recent advances focus on phase engineering (1T/2H coexistence), heteroatom doping (V, Co, Nb, P), and heterostructure formation (MoS_2_/MXene, MoS_2_/graphene, MoS_2_/black phosphorus) to overcome the intrinsic basal‐plane inertness of pristine 2H‐MoS_2_. These strategies enhance electrical conductivity, increase active‐site density, and promote interfacial charge transfer, resulting in significantly reduced overpotentials across acidic, alkaline, and neutral electrolytes. The electrolyte‐dependent performance trends observed in the table further emphasize that alkaline HER typically requires higher overpotentials due to sluggish water dissociation kinetics, which are mitigated by incorporating oxyphilic metals, such as Ni, Fe, Co, etc., that facilitate the Volmer step. Compared to MoS_2_, sesquichalcogenides and polychalcogenides offer distinct catalytic advantages that stem from their richer chalcogen content, structural complexity, and often metallic or semi‐metallic electronic character. As discussed above, MoS_2_ has long been studied as an earth‐abundant catalyst for HER because its edge sites possess near‐thermoneutral hydrogen adsorption energies, making them intrinsically active for proton reduction [201, 202]. However, the basal planes of MoS_2_ are largely inert in HER, and performance often depends on increasing edge exposure or phase transitions to 1T metallic domains to enhance conductivity and active‐site density [202]. In contrast, sesquichalcogenides such as FeS_2_, CoS_2_, and Ni_3_S_4_ possess non‐stoichiometric structures and a greater diversity of anion environments, which naturally generate higher densities of catalytically active sites across both edges and basal regions [203, 204]. Earlier studies on transition metal sulfosalt systems show smaller Tafel slopes and competitive overpotentials relative to certain MoS_2_ configurations due to the higher intrinsic electronic conductivity and multiple redox‐active centers in these systems, which facilitate charge transfer and proton adsorption more effectively than pristine MoS_2_ domains [204, 205], also reported in Table 2.

The table also reveals that for OER, the chalcogenide‐oxide or chalcogenide‐hydroxide heterostructures, such as CoOOH/Co_9_S_8_, Fe‐NiO/NiS_2_, NiCo LDH/NiCoS, outperform single‐phase materials. This improvement arises from in situ surface reconstruction under anodic potentials, where chalcogenides partially transform into catalytically active oxyhydroxide layers while retaining a conductive sulfide/selenide core. Vacancy engineering, heteroatom doping, and multimetal synergy (Ni‐Fe‐Co‐Mn systems) further optimize OER activity by tuning the adsorption energies of oxygen intermediates (OH*, O*, OOH*). Notably, several catalysts achieve overpotentials below 200 mV in alkaline media, underscoring the effectiveness of interfacial and defect engineering strategies. So it can be clearly stated here that the recent progress in catalytic performance is driven not merely by material selection but by rational catalyst design involving electronic modulation, phase control, and hierarchical heterostructuring. These insights provide a mechanistic framework explaining why certain ETM‐based chalcogenide systems outperform others.

Polychalcogenides further extend these catalytic tendencies by supporting dynamic surface states and defect‐rich frameworks that are beneficial for both HER and OER. Materials such as mixed metal selenides, tellurides, and higher‐order chalcogenide phases, such as CoSe_2_, NiSe_2_, mixed Co‐Fe selenides, etc., exhibit tunable band structures and abundant multivalent active sites, which can enhance intermediate adsorption and facilitate proton‐electron coupling in HER as well as multi‐electron transfer steps in OER. Recent studies suggest that transition metal selenides embedded in heterostructures display improved water adsorption and dissociation kinetics, leading to lower overpotentials compared to some MoS_2_ catalysts in alkaline conditions [248, 249]. The polychalcogenide framework also supports facile formation of catalytically active oxyhydroxide layers under anodic bias, which improves OER performance by combining a conductive chalcogenide core with in situ generated metal oxyhydroxide active phases [249]. While systematic comparisons are still emerging, these characteristics position sesqui and polychalcogenides as promising complements or even alternatives to MoS_2_ in electrocatalysis, particularly when durability, conductivity, and active‐site density are critical for performance across different pH conditions and water‐splitting reactions.

### 4.3 Sensing Applications

Chalcogenides are widely used in sensing applications due to their high sensitivity to light, heat, and gases. Their unique optical and electrical properties enable the detection of infrared radiation and environmental changes. These materials are ideal for gas sensors, photodetectors, and thermal imaging systems [250, 251, 252, 253]. A novel waveguide sensor was introduced, which used a silver island film (Ag‐IF) to enhance infrared absorption for detecting liquids and gases [254]. The sensor was built on a chalcogenide glass (Ge_28_Sb_12_Se_60_) rectangular waveguide and used lift‐off and oblique angle deposition methods, which made it easier to fabricate than traditional slot waveguides. It was observed that the 1.8 nm‐thick Ag film provided the best sensing performance. For ethanol detection at 1654 nm, the absorbance enhancement factor was >1.5, and for methane at 3291 nm, it was >2.3. The limit of detection (LoD) for CH_4_ was as low as 4.11% (1σ) with a response time of under 0.2 s. The sensor maintained good performance across the NIR and MIR ranges and showed reliable CH_4_ detection from shale gas samples. This design offered a compact, cost‐effective, and high‐performance platform for on‐chip sensing applications, bridging gas and liquid detection with fast response and improved light‐matter interaction.

**FIGURE 22:**
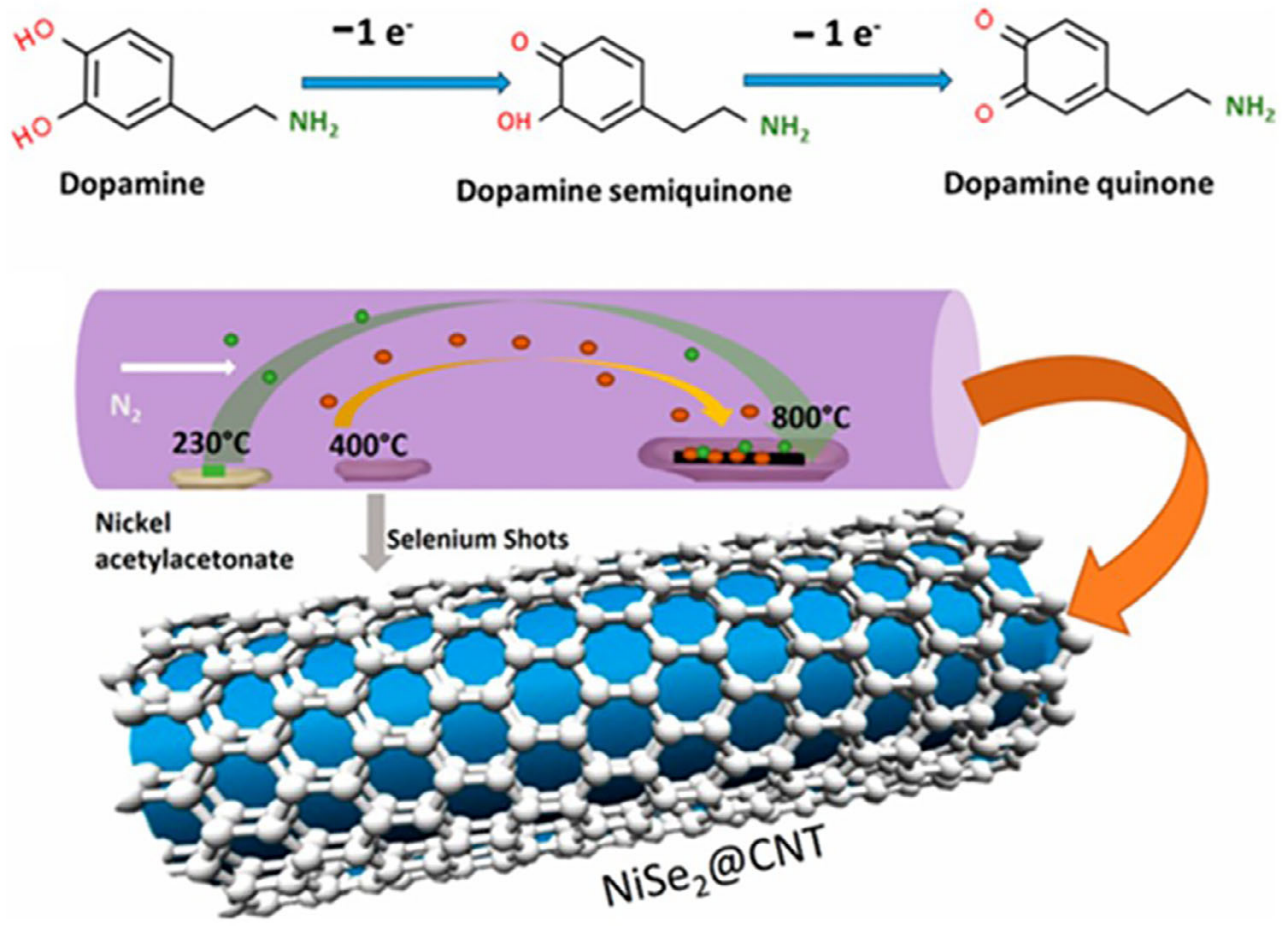
Synthesis scheme of the NiSe_2_@CNT composite and the oxidation scheme of DA through 1e^−^ electrode and 2e^−^ pathways. Reproduced with permission [255]. Copyright 2024, ACS.

A new electrochemical sensor has also been introduced for detecting dopamine using nickel selenide (NiSe_2_) nanostructures encapsulated in carbon nanotubes (CNTs) (see Figure 22) [255]. The sensor had a high sensitivity of 19.62 µA µM^−1^cm^−2^ and was able to detect dopamine as low as 5 nM by operating within a wide range from 5 nM to 640 µM. The CNTs enhanced the conductivity while NiSe_2_ boosts electrocatalytic activity, creating a synergistic effect that improved the sensor's response. This nonenzymatic sensor was also highly selective, even in the presence of interfering molecules like ascorbic acid, and worked effectively in real human tear samples, making it suitable for non‐invasive health monitoring. Tian et al. [256] focused on mercury chalcogenide colloidal quantum dots (QDs) for infrared (IR) light detection. It was observed that these QDs, like HgTe, HgSe, and HgS, can absorb light in a wide range from near‐IR (0.78 µm) to long‐wave IR (12 µm) by adjusting their size. Compared to traditional IR detectors (like InGaAs and HgCdTe), these QDs are cheaper, flexible, and can be processed in solution, which is ideal for portable or wearable IR devices. Notably, HgTe QDs have achieved performance similar to commercial detectors in the short‐ and mid‐IR ranges. Their applications included night vision, gas sensing, medical imaging, and temperature monitoring, as well as some devices that even used intra‐band transitions for longer wavelengths. It was concluded that Hg‐based QDs are excellent candidates for next‐generation, low‐cost, high‐sensitivity IR photodetectors, though concerns about toxicity and environmental safety still need addressing.

A novel method of assembling metal chalcogenide quantum dots (QDs) into gel structures using electrochemical techniques has also been reported. These QD gels retained the quantum properties of individual dots while forming porous, macroscopic 3D networks. Here, two gelation methods were used; among them, one used oxidation to form covalent dichalcogenide linkers, and the other relied on the metal ions to bind QDs through surface ligands. These gels were easy to form directly on device substrates, simplifying sensor fabrication, and the cadmium‐based QD gels showed high sensitivity in NO_2_ gas sensing, whereas the Pb‐doped gels offered improved response and recovery. Additionally, QD gels acted as efficient photocatalysts in organic synthesis due to their accessible surface area and electron transport. It was concluded that this new gelation strategy enables scalable, reusable, and tunable sensors and catalysts [257].

Chalcogenide glasses (such as As‐S‐Se) have been used in surface plasmon resonance (SPR) sensors, especially for biosensing [258]. A thin film of chalcogenide material was paired with graphene in a Kretschmann SPR configuration. The chalcogenide film enhances light‐matter interaction due to its high refractive index and photoresponsive nature. Simulations show that combining chalcogenide layers with graphene improves both sensitivity and signal‐to‐noise ratio (SNR) compared to conventional SPR sensors. The sensor is capable of detecting small changes in biological samples, like liver tissue, and can also be tuned to respond in the infrared region. The use of aluminum or gold layers further improves the device's plasmonic properties. This work supports the potential of chalcogenide materials in advanced biosensors for medical diagnostics, particularly in cases requiring high precision and IR‐range operation.

**FIGURE 23:**
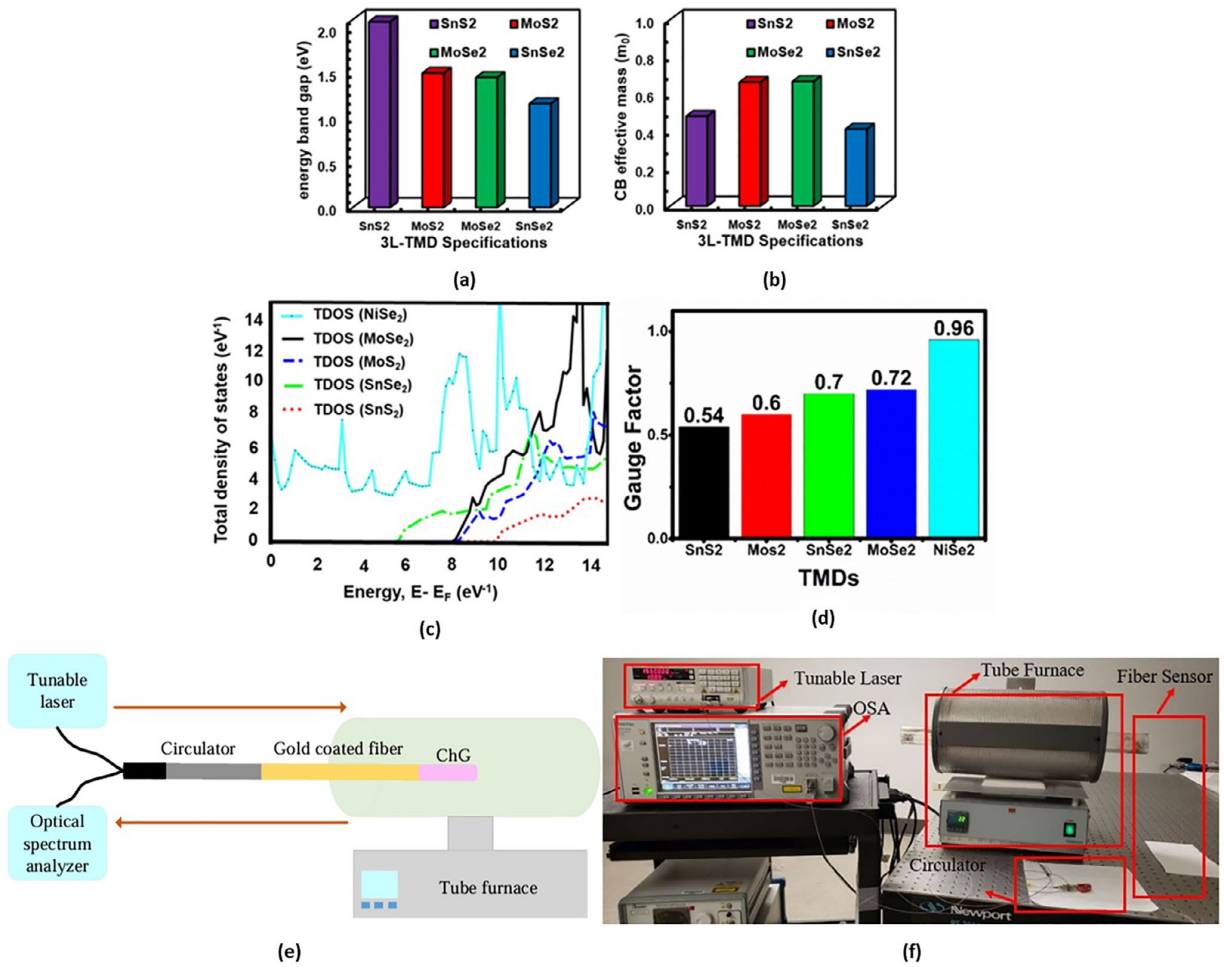
(a) Energy band gap of all TMDs, (b) conduction band average effective mass, (c) conduction band total density of states of tri‐TMDs, (d) comparison of experimentally extracted gauge factors of all TMDs strain sensors. Reproduced with permission [259]. Copyright 2022, Elsevier, (e) schematic of setup for testing the temperature performance of the fabricated sensors, and (f) a picture of the actual device characterization setup. Reproduced with permission [260]. Copyright 2021, MDPI.

In contrast, five different TMDs, i.e., MoS_2_, MoSe_2_, SnS_2_, SnSe_2_, and NiSe_2_, were compared as materials for flexible strain sensors [259]. Both experimental tests and DFT simulations found that NiSe_2_‐based sensors had the highest gauge factor, meaning they were most sensitive to strain. This was linked to the low bandgap of NiSe_2_ and the high density of states present in it, which enhanced the electron tunneling between nanoflakes when bent. The sensors were fabricated using vacuum filtration and encapsulated in PDMS, making them robust and wearable. This was the first time such a systematic correlation between theoretical electronic properties and experimental sensing performance had been made. This work established a framework for designing TMD‐based strain sensors, suggesting that selenide‐based TMDs offer the best performance due to their favorable band structure (see Figure 23a–d). Another group investigated the Ge_20_Se_80‐x_Bi_x_ (*x* ≤ 12) chalcogenide glasses for use in infrared and gamma radiation sensing. It was noted that as the bismuth (Bi) content increased, the glass became more denser and showed increased molar and free volume. However, the optical bandgap, compactness, and packing factor of the glass decreased, which indicated a softer and more disordered structure of the synthesized system. These glasses were promising for radiation shielding due to their gamma attenuation abilities, which were concluded by using Monte Carlo simulations in which the best shielding (lowest half‐value layer) was found at *x* = 12, with a value of 3.631 cm at 6 MeV energy. Their transparency in the IR range, combined with gamma shielding, made them suitable for applications in photonics, medical diagnostics, and nuclear protection.

Quaternary Ge‐Sb‐Se‐Te (GSST) thin films were developed using RF magnetron sputtering to explore their use in mid‐infrared sensors and nonlinear optics. Films were fabricated from Ge_19_Sb_17_Se_64‐x_Te_x_ (*x* = 5, 10, 15, 20) compositions. As Te content increased, the refractive index rose from 2.84 to 3.20 (at 1.55 µm), while the optical bandgap decreased from 1.41 eV to 1.05 eV. The best nonlinear refractive index was found in Ge_19_Sb_17_Se_56_Te_8_ film with n_2_ = 28 × 10^−18^ m^2^/W and figure of merit (FOM) = 0.11, showing high potential for telecom applications. Water contact angles ranged from 68–71°, with surface energies between 36–39 mJ/m^2^, suitable for sensor surface functionalization. Films remained amorphous even with partially crystalline targets. Overall, GSST films exhibit excellent IR transparency, thermal stability, and strong nonlinear optical properties, making them promising materials for photonic sensors and mid‐IR applications [261]. Some other chalcogenide glasse (ChG) caps on gold‐coated optical fibers have also been reported to be used as fiber‐optic temperature sensors, which are capable of withstanding extreme environments like nuclear reactors, shown in Figure 23e,f [260]. The sensor worked by detecting changes in optical reflectance during ChG's phase transition from amorphous to crystalline, which happens rapidly within 80–100 ns. Six ChG compositions were tested, and their crystallization temperatures (T_C_) ranged from 413°C (Ge_40_S_60_) to 605°C (Ge_30_S_70_). It was noted that their reflective power changed sharply at T_C_, enabling real‐time thermal monitoring between 440°C and 600°C. Measured refractive indices also shifted significantly, i.e., for Ge_40_S_60,_ it changed from 2.63 (amorphous) to 3.11 (crystalline) at 1550 nm, and its small footprint (2.125 mm^2^) further allowed array integration. The sensor was fabricated by using the dip‐coating technique and thermal evaporation technique and was tested in a high‐temperature furnace setup.

## 5 Challenges and Future Perspectives

ETM‐based chalcogenides have attracted strong scientific interest due to their impressive electrical, catalytic, and structural properties. As discussed above, their layered atomic arrangements, tunable band structures, and compatibility with van der Waals engineering make them promising candidates for next‐generation technologies. However, despite their potential, numerous challenges still limit their widespread use in large‐scale applications. A deeper understanding of these limitations and future research opportunities is essential for translating laboratory‐level discoveries into industrially meaningful technologies. This section discusses five major themes: scalability, environmental sustainability, structural and phase control, industrial adoption, and emerging research opportunities that must be critically evaluated to advance the field.

### 5.1 Scalability of Material Synthesis

Scalability remains one of the most critical challenges for ETM‐based chalcogenides, and addressing it is essential for translating laboratory discoveries into real commercial products. Current synthesis techniques, such as mechanical exfoliation, chemical vapor deposition (CVD), hydrothermal synthesis, and molecular beam epitaxy (MBE), have each enabled important scientific progress, but none of them fully meet industrial requirements for large‐area, high‐purity, and cost‐effective production. Mechanical exfoliation produces extremely high‐quality flakes but is inherently limited to small sizes and low throughput. CVD and MBE offer excellent control over thickness and crystal quality, yet their dependence on high temperatures, vacuum systems, and expensive precursors significantly increases operational cost and limits scalability. Meanwhile, hydrothermal or solvothermal methods can produce materials in larger batches, but the resulting crystals often show mixed phases or poor uniformity.

Recent advances in molten‐salt synthesis, plasma‐assisted growth, liquid‐phase exfoliation, and seeded crystal expansion demonstrate the possibility of low‐cost, continuous, and\ energy‐efficient fabrication. Roll‐to‐roll production, which has been successfully implemented for graphene, also shows potential for manufacturing large‐area chalcogenide films if the process can be adapted to ensure phase purity and defect control. Furthermore, machine‐learning‐driven optimization and real‐time diagnostics may soon enable automated tuning of growth parameters, improving reproducibility and minimizing waste. As these innovations are advancing, scalable and economically viable production of ETM‐based chalcogenides appears increasingly achievable, paving the way for widespread use in energy devices, sensors, and next‐generation electronics.

### 5.2 Environmental and Sustainability Challenges

Environmental sustainability is becoming an essential consideration for the development of advanced materials, and ETM‐based chalcogenides are no exception. Their synthesis often requires high temperatures, toxic precursors, energy‐intensive processes, and rare elements such as Mo, W, and Te. Additionally, solvent‐heavy procedures and acid‐based etching generate chemical waste streams that must be carefully managed. These factors raise environmental concerns and challenge the long‐term viability of industrial‐scale production.

However, recent efforts toward greener chemistry, waste minimization, and sustainable material sourcing suggest that environmentally responsible production is increasingly within reach. A particularly promising direction involves replacing hazardous chalcogen sources such as H_2_S or selenium oxides with safer options like thiourea, elemental sulfur, or organic precursors. These alternatives reduce toxicity and often allow reactions to proceed under milder conditions. Energy consumption can also be significantly reduced by adopting low‐temperature solution processing, photochemical synthesis, or plasma‐enhanced growth, which enable rapid reaction kinetics at reduced thermal budgets. Another important area of progress is substrate recycling. Many high‐value substrates used in chalcogenide synthesis, including sapphire and SiO_2_/Si wafers, can be reused multiple times with minimal performance impact, reducing both cost and waste generation. Life‐cycle assessments are increasingly being integrated into material development pipelines to quantify environmental footprints and identify stages with the greatest potential for improvement.

### 5.3 Structural Stability and Phase Control

Structural stability and phase control represent fundamental scientific challenges that directly influence the performance, reliability, and lifetime of ETM‐based chalcogenides. These materials often exhibit multiple metastable crystal phases with very small energy differences between them, making them highly sensitive to synthesis conditions, temperature changes, mechanical strain, and chemical environment. Unintended phase transitions such as conversion between the metallic 1T phase and the semiconducting 2H phase can dramatically affect electrical conductivity, catalytic activity, and optical behavior. Additionally, chalcogen vacancies, which are common during synthesis, may migrate under electrical bias or thermal stress, causing degradation or drift in device performance over time. Despite these challenges, significant progress has been made in achieving more reliable structural control. The use of encapsulation layers such as hexagonal boron nitride (h‐BN), graphene, or thin oxides has proven effective in stabilizing chalcogenide layers against oxidation and moisture exposure, enabling longer device lifetimes. Alloy engineering, such as forming Mo_1‐x_W_x_S_2_ or Ti_1‐x_Zr_x_X_2_ systems, offers another promising strategy by tuning the lattice environment to stabilize desired phases. Strain engineering, both intrinsic and substrate‐induced, provides additional control over phase stability and band structure, allowing tailored electronic properties without changing composition.

Future directions in this area are particularly exciting. In situ and operando characterization techniques are rapidly improving, enabling real‐time observation of atomic‐scale structural evolution during device operation. This will help us to identify failure mechanisms and design materials with improved robustness. Computational modeling and artificial intelligence are also becoming powerful tools for predicting stable phases under different temperature, doping, or strain conditions. Also, it can be said that materials with long‐term structural and phase stability will become increasingly feasible, greatly enhancing their utility in electronics, catalysis, sensors, and energy storage applications. Sustainability can be further strengthened by designing closed‐loop manufacturing processes, improving precursor utilization efficiency, and developing solvent‐free or water‐based synthesis pathways. These advances will not only reduce environmental impact but also make ETM‐based chalcogenides more attractive to industries that prioritize green manufacturing and regulatory compliance. As global attention increasingly shifts toward sustainable technologies, addressing environmental challenges will play a vital role in ensuring long‐term adoption.

### 5.4 Industrial Adoption and Economic Considerations

Bringing ETM‐based chalcogenides into real industrial use requires overcoming significant economic and engineering barriers. Although their unique properties make them promising for electronics, energy storage, and sensing, large‐scale industrial adoption remains limited by cost, manufacturing complexity, and integration challenges. Many existing synthesis methods rely on expensive equipment such as CVD and MBE reactors, which require specialized training, high energy input, and strict environmental controls. Additionally, the lack of standardized processes leads to inconsistencies in quality, making it difficult for industries to ensure device reliability and uniformity. Another major challenge is compatibility with existing industrial workflows, particularly in semiconductor fabrication, where the processes are finely tuned for silicon.

Advances in low‐temperature growth, scalable solution processing, and roll‐to‐roll deposition techniques promise to significantly reduce production costs and improve throughput. These methods could eventually enable direct integration of ETM‐based chalcogenides into flexible electronics, displays, and sensor systems without requiring high‐temperature steps that damage polymer substrates. Technical‐economic analysis and cost modeling are also emerging as essential tools for assessing commercial feasibility. These models help identify cost drivers such as precursor price, energy consumption, or substrate cost and allow optimization of processes accordingly. Collaboration between academic researchers, industrial engineers, and equipment manufacturers will be crucial for adapting laboratory techniques to manufacturing‐compatible formats.

### 5.5 Emerging Opportunities and Future Possibilities

Despite the challenges, ETM‐based chalcogenides offer a wide range of exciting opportunities for future technologies. Their atomic‐scale thickness, tunable band structures, strong spin–orbit coupling, and compatibility with van der Waals heterostructures make them ideal candidates for emerging research areas such as quantum technologies, neuromorphic computing, advanced catalysis, and next‐generation energy storage. Their ability to form heterostructures without lattice‐matching constraints opens pathways to unique material combinations that are not possible using conventional semiconductors. These heterostructures can enable novel functionalities such as gate‐tunable band alignments, interlayer excitons, and highly efficient charge separation for optoelectronic or catalytic applications.

In the field of sensing and nanoelectronics, ETM‐based chalcogenides hold promise for developing ultra‐sensitive chemical and biological sensors, high‐performance transistors, and flexible or transparent electronic systems. Their inherent 2D nature allows strong electrostatic gating and high on/off ratios, making them attractive for low‐power electronics and emerging logic applications. Similarly, the combination of large surface area, strong catalytic activity, and tunable active sites makes them promising materials for hydrogen evolution, CO_2_ reduction, nitrogen fixation, and other catalytic processes that support clean energy transitions. Many advancements can be expected from the integration of computational design, machine learning, and automated synthesis systems, which will accelerate the discovery of new ETM‐based structures and functionalities. The development of defect‐engineered materials, metastable phases, and multi‐functional heterostructures will also open new possibilities for device engineering. Future studies emphasizing long‐term stability, environmental safety, and manufacturability will play a critical role in ensuring that these materials transition beyond laboratory‐scale demonstrations and into real‐world technologies.

## 6 Conclusion

ETM‐based chalcogenides represent a fascinating class of materials with a wide variety of structures and useful properties. These compounds, formed by combining transition metals with elements like sulfur, selenium, or tellurium, offer a range of electronic behaviors from semiconducting to metallic. Their layered structures, ease of modification through doping and intercalation, and ability to undergo phase transitions make them extremely adaptable for cutting‐edge technologies. Throughout this review, we have explored the different categories of ETM‐based chalcogenides, such as disulfides, sesquichalcogenides, and polychalcogenides, as well as their crystal structures, including 1T, 2H, and 3R polytypes. The relationship between structure and function was emphasized, particularly in how coordination geometries affect conductivity, catalytic activity, and optical behavior. The practical applications of these materials continue to grow, including their use in transistors, sensors, batteries, solar cells, superconductors, and catalysts. Techniques such as chemical doping, intercalation, and strain engineering have allowed us to customize their properties for specific needs. Additionally, advances in synthesis methods now enable the formation of nanosheets and heterostructures that improve performance and expand functionality. Defects, polymorphism, and stacking order play crucial roles in determining a material's performance, especially in fields like memory storage and energy devices. The ongoing discovery of new phases and the ability to manipulate these materials at the atomic level have opened exciting research directions.

In summary, this review has systematically discussed the structural characteristics, electronic behavior, catalytic functionality, and application potential of 2D early transition metal chalcogenides, with particular emphasis on transition metal dichalcogenide‐based systems. Through a critical assessment of recent experimental and theoretical studies, the manuscript highlights how crystal structure, phase composition, dimensionality, defect chemistry, and interfacial engineering collectively govern the performance of these materials in electronic, optoelectronic, catalytic, and energy‐conversion applications. Beyond consolidating recent advances, Section 5 has identified several key scientific and technological bottlenecks that must be addressed to enable practical deployment. One major challenge lies in the scalable and reproducible synthesis of high‐quality chalcogenide materials with precise control over thickness, phase, stoichiometry, and defect density. Although laboratory‐scale techniques such as chemical vapor deposition, hydrothermal synthesis, and liquid‐phase exfoliation have achieved impressive material quality, translating these approaches to wafer‐scale production while maintaining uniformity and cost‐effectiveness remains nontrivial. In addition, phase instability, environmental degradation, and long‐term operational reliability, particularly under electrochemical or high‐bias electronic conditions, continue to limit real‐world applications.

Another critical issue emphasized is the incomplete understanding of interfacial phenomena in both electronic devices and catalytic systems. Contact resistance, Schottky barrier formation, charge trapping, and interlayer coupling strongly influence device performance, yet their atomic‐scale mechanisms remain insufficiently resolved. Similarly, in electrocatalysis, the precise roles of edge sites, basal plane activation, heteroatom doping, and phase engineering in governing reaction pathways and kinetics require further mechanistic clarification. Addressing these gaps will demand integrated experimental‐theoretical approaches, combining advanced in situ characterization techniques with first‐principles simulations. Looking forward, future research directions highlighted in this review include the rational design of heterostructures, controlled defect and dopant engineering, and the exploration of underrepresented material classes such as sesqui‐ and polychalcogenides. These materials offer distinct electronic structures, richer redox chemistry, and potentially superior catalytic activity compared to conventional dichalcogenides, yet remain largely unexplored in applied contexts. Moreover, the convergence of transition metal chalcogenides with emerging platforms such as flexible electronics, neuromorphic computing, photo‐electrocatalysis, and multifunctional energy devices represents a promising avenue for next‐generation technologies.

Overall, by integrating recent progress with a critical analysis of existing limitations and future opportunities, this review provides a coherent framework for understanding the current state of early transition metal chalcogenide research. It is anticipated that the insights and perspectives presented here will guide future investigations and accelerate the development of robust, scalable, and high‐performance chalcogenide‐based systems for advanced electronic and catalytic applications.

## Conflicts of Interest

The authors declare no conflicts of interest.
